# Utility of high-sensitivity cardiac troponin I normalized to left ventricular mass in detection of stable coronary artery disease

**DOI:** 10.1038/s44161-026-00837-z

**Published:** 2026-07-30

**Authors:** Sina Fathieh, Michael P. Gray, Owen Tang, Katharine A. Bate, Stephen T. Vernon, Elijah Genetzakis, Soloman Saleh, Gillian Murtagh, Jean Y. Yang, Collin Tran, David R. Sullivan, Paul Bonnitcha, David S. Celermajer, Louise Cullen, Ravinay Bhindi, Jamil Mayet, Stephen J. Nicholls, Stuart M. Grieve, Gemma A. Figtree

**Affiliations:** 1 School of Medical Sciences, University of Sydney, Camperdown, New South Wales Australia; 2 National Heart and Lung Institute, Imperial College London, London, UK; 3 Division of Cardiovascular Medicine, University of Oxford, Oxford, UK; 4 Core Diagnostics, Abbott Laboratories, Abbott Park, IL USA; 5 School of Mathematics and Statistics, The University of Sydney, Camperdown, New South Wales Australia; 6 Sydney Precision Data Science Centre, Sydney, New South Wales Australia; 7 NHMRC Clinical Trials Centre, University of Sydney, Sydney, New South Wales Australia; 8 Department of Chemical Pathology, Royal Prince Alfred Hospital, Camperdown, New South Wales Australia; 9 NSW Health Pathology, Sydney, New South Wales Australia; 10 Department of Cardiology, Royal Prince Alfred Hospital, Sydney, New South Wales Australia; 11 Heart Research Institute, Sydney, New South Wales Australia; 12 Emergency and Trauma Centre, Royal Brisbane and Women’s Hospital, Brisbane, Queensland Australia; 13 Department of Cardiology, Royal North Shore Hospital, Sydney, New South Wales Australia; 14 Imperial College Healthcare NHS Trust, London, UK; 15 Victorian Heart Institute, Monash University, Melbourne, Victoria Australia; 16 Lumus Imaging, St George Private Hospital, Kogarah, New South Wales Australia

**Keywords:** Diagnostic markers, Ischaemia

## Abstract

Coronary artery disease often develops silently before myocardial infarction, and current risk-based prevention pathways miss some individuals with clinically important disease, particularly those without standard modifiable cardiovascular risk factors. High-sensitivity cardiac troponin I is a blood biomarker of low-level myocardial injury, but its relationship to silent coronary atherosclerosis remains incompletely understood. Here we show, in 1,613 adults undergoing coronary computed tomography (CT) imaging, that low-level high-sensitivity cardiac troponin I is associated with clinically actionable coronary disease, defined by coronary calcium score ≥100, and with quantitative CT markers of plaque burden. A threshold of 2.3 ng l^−1^ enriched for actionable disease, especially in individuals without standard risk factors. Normalizing troponin for myocardial mass, age and renal function improved prediction and provided mechanistic insight into baseline troponin elevation in chronic coronary syndromes. A two-stage strategy using troponin before CT imaging increased diagnostic yield and reduced the number of scans required. Prospective validation is warranted before clinical implementation.

## Abstract (web summary)

Fathieh et al. investigate the clinical value of a pre-screening strategy based on high-sensitivity troponin levels to identify patients at risk of coronary artery disease and in need of imaging diagnosis, even in the absence of traditional risk factors.

## Main

Atherosclerotic coronary artery disease (CAD) is the causal pathophysiology in the vast majority of acute myocardial infarction (MI) presentations and associated sudden cardiac deaths; however, it commonly goes undetected for years before an event. Preventative strategies have been successful in identifying and targeting standard modifiable cardiovascular risk factors (SMuRFs)—including dyslipidemia, hypertension, smoking and diabetes—known at a population level to increase the probability of developing atherosclerosis. However, there remains considerable variation in individual responses to these factors, with 10–27% of first-time heart attack patients developing CAD-related acute MI despite no risk factors meeting currently accepted primary prevention thresholds, and others having a full ‘suite’ of risk factors with no evidence of the disease process^1–4^. Evidence that intensive low-density lipoprotein (LDL) cholesterol-lowering can halt the progression of CAD^5–7^ and stabilize the plaque phenotype^7,8^, dramatically reducing MI^9^, makes it critical to develop new tools and pathways for early disease detection. This would allow patients and their physicians to move beyond probability to treating the causal disease. This is the goal of our international consortium CAD Frontiers (https://cadfrontiers.com.au)^10^, and is consistent with the recent ‘call to arms’ by the Lancet Commission for Rethinking CAD.

The burden of chronic CAD (patients with detectable CAD on imaging, with and without symptoms who have not had an acute event) and the opportunity for effective, targeted CAD treatment to prevent heart attacks has also been highlighted in the Swedish Cardiopulmonary Bioimage Study^11^ where among more than 25,000 randomly selected individuals between the ages of 50 and 64 who were screened with coronary angiography CT (CCTA), chronic CAD was common (42.1%), high-grade stenosis was relatively prevalent (5.2%), and severe CAD was found in 1.9% of their population. However, such an approach is not considered practical or appropriate for screening the entire community^12^. These findings underscore the urgent need for blood-based biomarkers to bridge the gap, improving appropriate triage to noninvasive coronary imaging, with the potential to increase the scale and equity of CT-based screening for subclinical CAD.

Troponin is a well-established clinical diagnostic marker for acute coronary syndrome (ACS) and a prognostic marker of heart failure development in healthy, asymptomatic individuals^13^. With advancements in high-sensitivity cardiac troponin (hs-cTn) assays, its potential association with CAD in suspected chronic coronary syndrome populations is now being explored. Some studies have linked hs-cTn with coronary atherosclerosis in the setting of stable angina^14^ and end-stage renal failure^15^. Very recently, a meta-analysis by the CAPRICE Co-investigators published in the *Journal of the American College of Cardiology*^16^ used data from 15 cohorts, comprising 62,150 participants without prior cardiovascular disease (CVD) and demonstrated that measurement of hs-cTn results in a modest improvement in the prediction of first-onset CVD^16^. This supports the potential role of hs-cTn for risk stratification. However, data on the value of hs-cTn for detecting atherosclerotic CAD before an event, as called for by the Lancet Commission^12^ and a recent patient/consumer manifesto^17^ is not available. In this study, we aimed to investigate the utility of high-sensitivity cardiac troponin I (hs-cTnI) in detecting CT-defined clinically actionable CAD, primarily defined by coronary artery calcium burden, while also examining its relationship with secondary CCTA-derived measures of plaque burden and myocardial mass.

## Results

### Patient characteristics and hs-cTnI distribution

Of the 1,613 patients, 473 had CAD classified as clinically actionable. These participants were, on average, older, more frequently male and more likely to have three or four SMuRFs (Table 1). All participants in the study with measured hs-cTnI had levels above the limit of detection (LoD) with a right-skewed distribution (Shapiro–Wilk *W* = 0.14, *P* < 0.001), a median of 2.0 ng l^−1^ and a 99th percentile of 87.3 ng l^−1^. The 99th percentile is classically derived from ‘healthy’ age- and sex-appropriate community patients. However, this likely includes a substantial number with chronic CAD. The 99th percentile value for individuals without detectable coronary artery calcification in the BioHEART cohort was 32 ng l^−1^ (versus 87.3 ng l^−1^ for the entire cohort).

**Table 1:** Baseline demographic data

	Overall (*n* = 1,613)	CACS 0 (*n* = 638)	CACS 1–99 (*n* = 502)	CACS 100+ (*n* = 473)
Age	61.4 (11.8)	54.8 (11.2)	62.6 (9.74)	69.2 (9.32)
Male	923 (57.2%)	290 (45.5%)	300 (59.8%)	333 (70.4%)
BMI	26.3 [13.1, 51.3]	26.0 [15.8, 50.8]	26.8 [13.1, 49.5]	26.4 [16.4, 51.3]
European	1,377 (85.4%)	511 (80.1%)	433 (86.3%)	433 (91.5%)
SMuRFs				
0	341 (21.1%)	205 (32.1%)	88 (17.5%)	48 (10.1%)
1	664 (41.2%)	294 (46.1%)	214 (42.6%)	156 (33.0%)
2	441 (27.3%)	115 (18.0%)	146 (29.1%)	180 (38.1%)
3	146 (9.1%)	24 (3.8%)	47 (9.4%)	75 (15.9%)
4	21 (1.3%)	0 (0%)	7 (1.4%)	14 (3.0%)
Diabetes	139 (8.6%)	34 (5.3%)	40 (8.0%)	65 (13.7%)
Hypertension	657 (40.7%)	178 (27.9%)	231 (46.0%)	248 (52.4%)
Smoking history	315 (19.5%)	78 (12.2%)	94 (18.7%)	143 (30.2%)
Dyslipidemia	957 (59.3%)	306 (48.0%)	310 (61.8%)	341 (72.1%)
Symptomatic	878 (54.4%)	376 (58.9%)	272 (54.2%)	230 (48.6%)
hs-cTnI (ng l^−1^)	2.00 [0, 541]	1.60 [0, 174]	2.00 [0, 117]	2.80 [0, 541]
Creatinine (mg dl^−1^)	70.9 [25.9, 889]	69.3 [28.4, 664]	70.6 [25.9, 457]	72.7 [26.1, 889]
LDL (mmol l^−1^)	2.95 (0.977)	3.11 (0.949)	3.00 (0.980)	2.67 (0.952)
HDL (mmol l^−1^)	1.33 (0.418)	1.37 (0.445)	1.31 (0.410)	1.29 (0.381)
CRP (mg dl^−1^)	1.17 [0.0200, 610]	1.05 [0.0250, 92.4]	1.20 [0.0200, 141]	1.26 [0.0550, 610]
CACS (AU)	11.5 [0, 8,340]	0 [0, 0]	23.3 [0.110, 99.5]	368 [100, 8340]
Gensini	3.50 [0, 110]	0 [0, 20.0]	5.00 [0.500, 33.0]	17.5 [2.50, 110]
NCPS	2.50 [0, 86.0]	0 [0, 37.0]	3.50 [0, 50.0]	11.5 [0, 86.0]

HDL, high-density lipoprotein; CRP, C-reactive protein; NCPS, noncalcified plaque score.

Left ventricle myocardial mass (LVM) followed a normal distribution (mean ± s.d. = 172 ± 31 g) in men and 122 ± 20 g in women, displaying a strong correlation with hs-cTnI (Spearman’s correlation coefficient (*ρ*_s_) = 0.32; *P* < 0.001 – log-linear relationship is shown in Extended Data Fig. 2), as well as weight, height and body surface area (BSA) (*ρ*_s_ = 0.7, 0.67, 0.76, respectively; *P* < 0.05).

### Correlations with demographics and clinical measures

Hs-cTnI concentration was associated with age, weight and height with *ρ*_s_ values of 0.27, 0.19 and 0.18, respectively (*P* < 0.001). Other associations included estimated glomerular filtration rate, number of SMuRFs and C-reactive protein (*ρ*_s_ = −0.27, 0.14 and 0.08, respectively; *P* < 0.001).

**Fig. 1:**
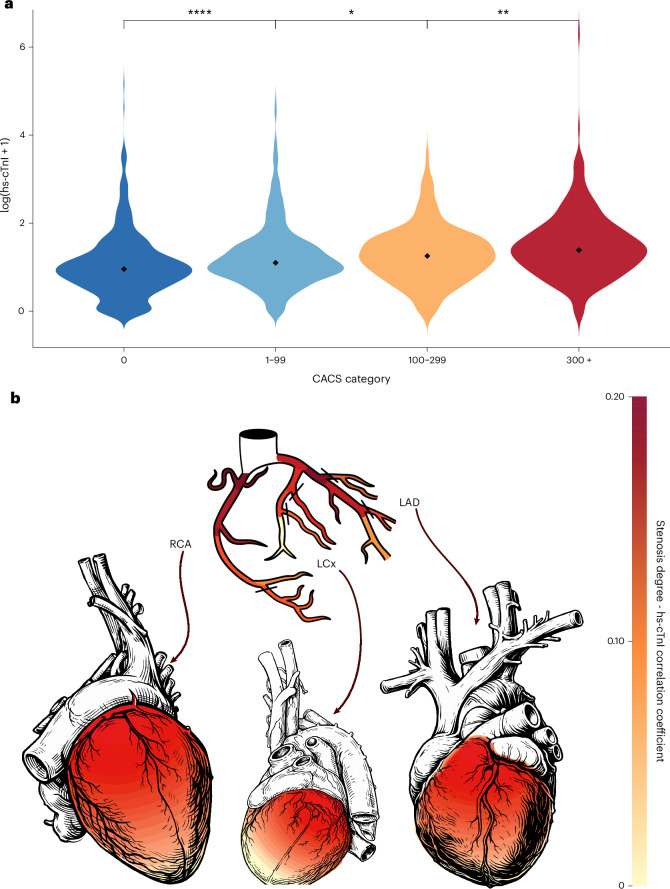
Effect of coronary artery disease burden on the background hs-cTnI levels. **a**, Distribution of hs-cTnI across CACS categories. Violin plots show the distribution of log-transformed hs-cTnI concentrations, calculated as log(hs-cTnI + 1), according to CACS category. Black diamonds indicate group medians. Group sizes were: CACS 0, *n* = 638; CACS 1–99, *n* = 502; CACS 100–299, *n* = 196; and CACS ≥ 300, *n* = 277. Pairwise comparisons were performed using two-sided Wilcoxon rank-sum tests with Holm adjustment for multiple comparisons: CACS 0 versus 1–99, *P* = 1.6 × 10^−^^7^; CACS 1–99 versus 100–299, *P* = 0.027; and CACS 100–299 versus ≥300, *P* = 0.009. **b**, Heatmap reflecting the correlation coefficient of plaque burden and hs-cTnI level for each segment. The relationship between segmental plaque burden and hs-cTnI is progressively stronger in proximal segments that supply a larger downstream myocardial mass. LAD, left anterior descending artery; LCx, left circumflex artery; RCA, right coronary artery. *P < 0.05, **P < 0.01, ***P < 0.001.

Hs-cTnI was significantly higher in patients with clinically actionable CAD versus those with a coronary artery calcium score (CACS) of 1–99 (1.5-fold, *P* < 0.001) and those with CACS = 0 (1.8-fold, *P* < 0.001) (Fig. 1a). There was a significant association between hs-cTnI and CACS (*ρ*_s_ = 0.27; *P* < 0.001), Gensini score (*ρ*_s_ = 0.26; *P* < 0.001) and noncalcified plaque score (*ρ*_s_ = 0.21; *P* < 0.001).

Hs-cTnI was significantly correlated with the total number of coronary artery segments with any detectable plaque (*ρ*_s_ = 0.25; *P* < 0.001). Given the association with the degree of CAD and myocardial mass, we explored the possibility that the hs-cTnI with CAD may be stronger with more ‘downstream’ myocardium. Spearman’s correlation assessing the segment location and the plaque burden (stenosis in each segment) against hs-cTnI revealed a stronger correlation in the proximal segments, as schematically shown in Fig. 1b, consistent with an important role for the downstream mass of myocardium from the contributing atherosclerotic lesion.

### Diagnostic performance

Hs-cTnI was significantly higher in patients with clinically actionable CAD (CACS ≥ 100) than in those with CACS = 1–99 and those with CACS = 0 (median 2.8 versus 2.00 and 1.60, *P* < 0.001). There was a significant association between hs-cTnI and CACS, Gensini score and noncalcified plaque score (*ρ*_s_ = 0.27, 0.26 and 0.21, respectively; *P* < 0.001). Receiver operating characteristic (ROC) curve analysis indicated a modest performance of hs-cTnI levels in classifying clinically actionable CAD (area under the ROC curve (AUC) = 0.64, 95% confidence interval (CI) 0.61–0.67).

An optimal threshold was determined at 2.35 ng l^−1^ (sensitivity = 0.60, specificity = 0.65), closely aligning with a cutoff of 2.32 ng l^−1^ derived from 10,000 bootstrapped resamples, maximizing Youden’s index (Extended Data Fig. 3). Subgroup analyses yielded specific optimal thresholds for men (2.38 ng l^−1^; AUC = 0.62, 95% CI 0.58–0.66), women (2.31 ng l^−1^; AUC = 0.62, 95% CI 0.57–0.67) and SMuRF-less individuals (2.29 ng l^−1^; AUC = 0.66, 95% CI 0.59–0.73), as shown in Extended Data Fig. 4. The threshold for the SMuRF-less group (2.29 ng l^−1^) showed more promising performance characteristics, with a sensitivity of 0.69 and a negative predictive value (NPV) of 0.92. Full details are provided in Table 2. For pragmatic application, given the assay’s error rate at lower concentrations, we used a rounded cutoff of 2.3 ng l^−1^ for the rest of our analysis.

**Table 2:** Optimal cutoff points for overall, male, female and SMuRF-less subgroups to maximize Youden’s Index

Group	Cutoff	Youden’s	Sensitivity	Specificity	PPV	NPV	Accuracy	OR (95% CI)
All	2.32	0.25	0.60	0.65	0.41	0.79	0.64	2.77 (2.23–3.46)
Male	2.38	0.20	0.64	0.55	0.45	0.73	0.59	2.28 (1.73–3.02)
Female	2.31	0.23	0.48	0.75	0.33	0.85	0.70	2.85 (1.94–4.19)
SMuRF-less	2.29	0.34	0.69	0.65	0.24	0.92	0.66	4.18 (2.21–8.26)

**Fig. 2:**
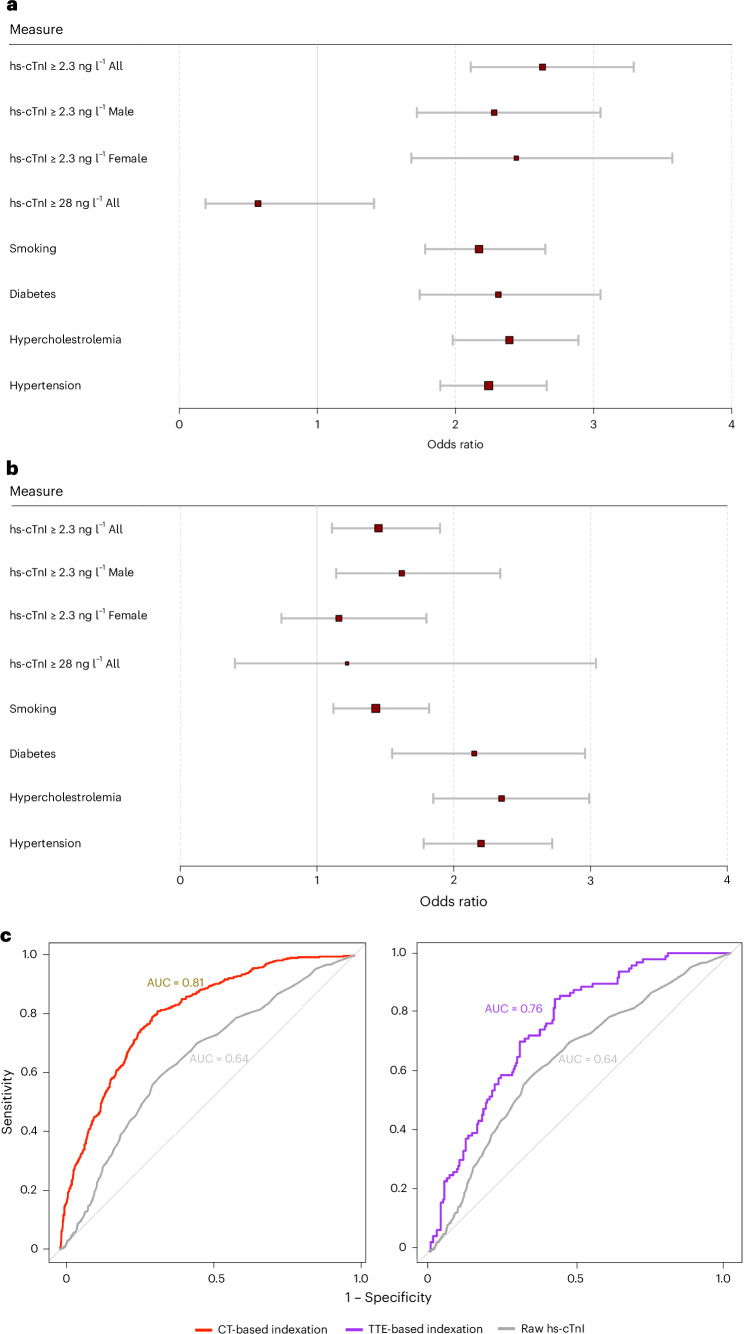
Performance of hs-cTnI in detecting clinically actionable coronary artery disease. **a**, Univariable logistic regression ORs with 95% CI for the prediction of clinically actionable CAD. Points represent ORs from univariable logistic regression models, and horizontal bars represent 95% CI. CACS ≥ 100, analyzed in *n* = 1,613 participants, including *n* = 473 with CACS ≥ 100 and *n* = 1,140 with CACS < 100. **b**, CACS percentile ≥75th percentile, relative to age- and sex-adjusted Multi-Ethnic Study of Atherosclerosis reference data. Dichotomized hs-cTnI was defined as ≥2.3 versus <2.3 ng l^−1^ and compared with traditional cardiovascular risk factors entered as binary variables: smoking history, diabetes, hypercholesterolemia and/or statin use and hypertension. All univariable predictor analyses in **a** and **b** used *n* = 1,613 participants; sex-stratified hs-cTnI analyses included *n* = 923 male and *n* = 690 female participants. Horizontal bars represent 95% CI; the vertical dashed line indicates OR = 1.0, representing the null effect. **c**, Left: ROC curves comparing raw hs-cTnI with hs-cTnI indexed using CT-derived myocardial mass for prediction of CACS ≥ 100 in *n* = 1,554 participants. Right: ROC curves comparing raw hs-cTnI with hs-cTnI indexed using TTE-derived myocardial mass in *n* = 359 participants. AUC values are shown for each model.

As shown in Table 2, a hs-cTnI cutoff of 2.3 ng l^−1^ for predicting clinically actionable CAD demonstrated similar efficacy in men and women, but had the greatest prognostic value in SMuRF-less individuals (odds ratio (OR) ≈ 4). Notably, in comparison with the use of the 2.3 ng l^−1^ cutoff, a conventional 28 ng l^−1^ MI detection threshold was not able to effectively classify individuals with actionable CAD. Notably, hs-cTnI levels >2.3 ng l^−1^ showed lower performance when compared to the high CACS percentile (≥75th percentile), a relative marker of CAD risk adjusted for age and sex (Fig. 2). This is consistent with hs-cTnI reflecting the absolute burden of atherosclerotic CAD rather than its susceptibility.

### Multivariable model development for prediction of clinically actionable CAD

1. Model 1: hs-cTnI alone. hs-cTnI above the identified threshold of 2.3 ng l^−1^ alone demonstrated moderate predictive power for CACS > 100, with an OR of 2.77 (95% CI 2.23–3.46), corresponding to modest discrimination (AUC 0.624; 95% CI 0.598–0.651). This model serves as a reference point for subsequent adjustments.
2. Model 2: integrating myocardial mass. Incorporating myocardial mass significantly improved the predictive accuracy. The interaction between elevated hs-cTnI and myocardial mass was statistically significant (interaction *P* = 0.012), with elevated hs-cTnI showing an enhanced OR of 8.87 (95% CI 3.38–23.5). This suggests myocardial mass substantially modifies the relationship between hs-cTnI levels and CAD presence, and could be incorporated to increase its clinical utility. Model discrimination improved modestly but significantly compared to Model 1 (*Δ*AUC = 0.020, *P* = 0.01). In univariate analysis, CT-derived LVM alone showed modest discrimination for clinically actionable CAD (OR = 1.01, *P* < 0.01; AUC = 0.55, CI 0.52–0.58), confirming it as an independent, although weaker, predictor compared with hs-cTnI and reinforcing the added value of LVM-indexed hs-cTnI. It is worth noting that in a post-hoc sensitivity analysis, given the strong correlation between sex and LVM, a model incorporating the interaction between hs-cTnI and sex demonstrated similar discrimination to the corresponding hs-cTnI-by-LVM model (ROC AUC 0.65 [95% CI 0.630–0.686] versus 0.65 [0.615–0.674]; DeLong *P* = 0.13) while the LVM interaction model demonstrated superior overall model fit, with lower Akaike information criterion (1,828.2 versus 1,848.1) and BIC (1,849.7 versus 1,869.6). In a subgroup analysis of men, LVM remained as a significant independent variable. Both sex and body mass index (BMI) become insignificant in a model that also includes LVM, indicating that in the absence of LVM measurements, surrogate markers such as sex may be useful, but myocardial mass captures information beyond crude sex stratification alone.
3. Model 3: accounting for renal clearance. Adding serum creatinine as a measure of renal clearance, while accounting for the impact of age and sex, further enhanced the model (*Δ*AUC = 0.16, *P* < 0.001), indicating an independent predictive value of renal function, as well as the known effect of age and sex. We refer to this model as the ‘indexed-troponin model’. Results of the 3 model comparisons are summarized in Table 3. Regression coefficients for Model 3, both with dichotomized and also continuous hs-cTnI are included in Supplementary Table 1. Sensitivity analyses using alternative definitions of CAD (CACS > 0 and CACS percentile ≥75th) yielded directionally similar associations between hs-cTnI and plaque burden (Supplementary Table 2).

**Table 3:** Model performance comparison

	CACS >100 ~	Sensitivity	Specificity	PPV	NPV	AUC %	hs-cTnI OR (CI)
Raw	hs-cTnI > 2.3	0.65	0.59	0.41	0.79	62.4	2.77 (2.23–3.46)
Model 1	hs-cTnI > 2.3 × LVM	0.64	0.60	0.41	0.79	64.4**	8.87 (3.38–23.5)
Model 2	hs-cTnI > 2.3 × LVM + hs-cTnI > 2.3 × Cr	0.64	0.60	0.41	0.79	65.6*	13.6 (4.24 – 44.4)
Model 3	hs-cTnI > 2.3 × LVM + hs-cTnI > 2.3 × Cr + Age:Cr + Age:LVM + sex × LVM	0.76	0.75	0.56	0.88	81***	4.65 (1.34–16.4)

Cr, creatinine.

*P < 0.05; **P < 0.01; ***P < 0.001. ~ denotes ‘regressed on’, with the outcome variable on the left and the predictor variable(s) in each row within the column.

#### Subgroup analysis based on transthoracic echocardiography-derived LVM

Given that CT measures of myocardial mass are not pragmatically available (which is included here as a mechanistic insight), we examined the performance of our latent score in a clinical application framework when derived using an echocardiographic measure of LVM (left ventricle septal wall thickness, indexed to BSA). Echocardiographic data were available for only 362 patients in whom transthoracic echocardiography (TTE)-derived LVM had a moderate correlation with CT-derived LVM (Pearson’s *ρ* = 0.74, *P* < 0.001). In this subcohort, AUC showed a promising improvement from baseline of 0.64 to 0.76 [0.70–0.811] for the overall cohort and 0.77 [0.65–0.89] for the SMuRF-less population (Fig. 2c).

#### Subgroup analysis based on symptom status

In this pragmatic cohort study, 55% of patients had been referred for stable symptoms, and 45% were asymptomatic; ‘screening’ studies were patient-driven and not reimbursed by the government. Of interest, a higher plaque burden was observed in the asymptomatic subcohort, as shown in Supplementary Table 3. We then performed a sensitivity analysis to test the predictive performance of the adjusted hs-cTnI in the asymptomatic subcohort. It demonstrated comparable performance in both groups (ROC AUC in symptomatic patients was 81.5% [78.3–84.6] versus 81.8% [78.5–81.8] in asymptomatic patients).

### Utility in clinical practice

**Fig. 3:**
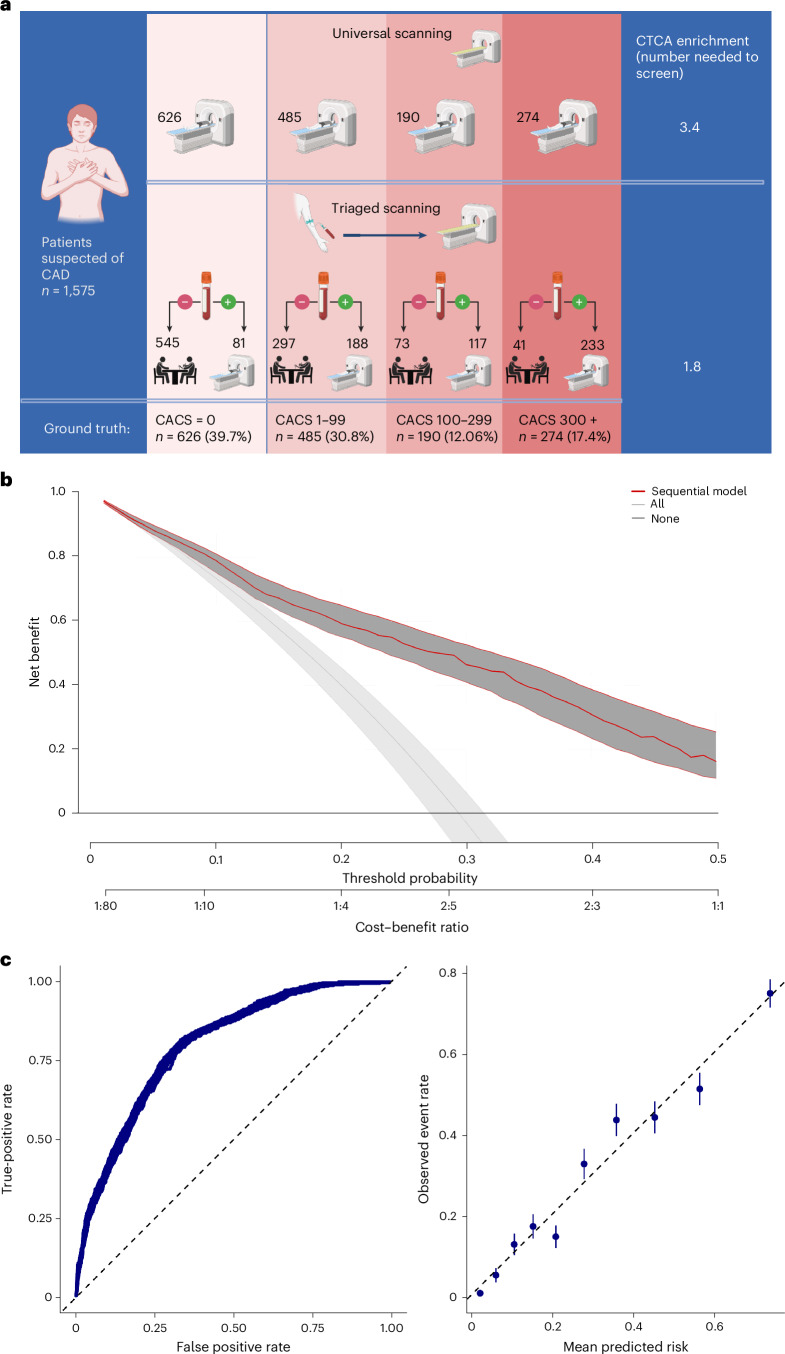
Utility of hs-cTnI in a two-stage screening program for detection of clinically actionable coronary artery disease. **a**, Comparison of universal scanning versus indexed-troponin model triaging for detection of clinically actionable CAD. Flow diagrams illustrate the classification outcomes of two screening approaches in 1,575 patients suspected of CAD, with measured hs-cTnI, LVM and creatinine for modeling. Upper: Universal screening CT CACS for all patients. Lower: hs-cTnI triage approach: initial risk stratification using the adjusted hs-cTnI model, followed by targeted CT CACS only in patients screening positive. The sequential approach enriches CCTA by improving the NNS to detect one clinically actionable CAD. **b**, Decision curve analysis comparing universal versus sequential screening strategies for clinically actionable CAD detection. Lines show median net benefit across posterior draws, and shaded bands show the 2.5th to 97.5th percentile interval. Net benefit is plotted against the threshold probability (upper axis) and the corresponding cost–benefit ratio (lower axis). The sequential screening strategy (red line)—using an initial adjusted hs-cTnI model to triage patients for subsequent CT CACS—is shown alongside ‘treat all’ and ‘treat none’ strategies (gray and black lines, respectively). The error region surrounding the sequential model curve represents the 95% CI derived from 1,000 bootstrap resamples. **c**, Internal validation of the Bayesian hs-cTnI model. Left: 100 ROC curves (light blue lines) were generated from random posterior draws of the Bayesian model, with the mean ROC curve overlaid (dark blue line). The dashed diagonal represents chance discrimination (AUC = 0.5). The posterior median AUC was 0.799, demonstrating stable discriminative performance after accounting for measurement error at low hs-cTnI concentrations. Right: calibration plot by deciles of predicted risk. Observed event rates are plotted against mean predicted probabilities for each decile of predicted risk. Points represent decile-level observed proportions derived from participant-level predictions; vertical error bars represent ±1 s.e. of the observed event proportion, calculated as √[*p*(1−*p*)/*n*] in each decile, where *p* is the observed proportion of participants who experienced the event within that decile and *n* is the number of participants in that decile. Calibration was assessed across ten deciles of predicted risk in *n* = 1,575 participants with complete data for the Bayesian model covariates. Panel **a** created in BioRender; Figtree, G. https://biorender.com/ipltliw (2026).

Given the increasing emphasis on early detection and treatment of CAD before an acute event^3,12,18,19^, we directly compared the clinical utility of two screening strategies: universal screening with CT coronary artery calcium scoring (CT CACS) for all patients versus a two-stage sequential screening approach, guided initially by an adjusted multivariable predictive model, triaging individuals for a CT CACS (Fig. 3c). Universal screening of the 1,575 participants with all data required for modeling inherently identified 464 of 1,575 patients with clinically actionable CAD (CACS > 100), but required imaging of the entire cohort. Conversely, diagnostic efficiency markedly improved by the use of adjusted hs-cTnI to triage individuals for CT CAC, nearly doubling the yield of clinically actionable CAD per CT performed (0.57 versus 0.29 cases per scan). The number needed to screen (NNS) with CT CACS to detect a single case of clinically actionable CAD using the indexed-troponin model to triage patients was 1.8, compared to 3.4 in the ‘take all’ approach. Although a higher number of patients are initially screened using a noninvasive test, we obtain a significantly higher positive imaging yield (Table 4).

**Table 4:** Comparison of CAD screening strategies

Metric	Universal screening	Sequential screening
Sensitivity (%)	100	76
Specificity (%)	—	75
Scans performed	1,575	619
Scans reduction (%)	0	61
Missed diagnosis (%)	0	114
NNS	3.4	Stage 1 = 4.47 Stage 2 = 1.8
Diagnostic yield (cases per scan)	0.29	0.57
LR+	—	3.04
LR−	—	0.32
DOR	—	9.5
Bayesian PPV	—	0.56
Bayesian NPV	—	0.88

DOR, diagnostic odds ratio; LR+, positive likelihood ratio; LR−, negative likelihood ratio.

Bayesian analyses demonstrated a positive predictive value (PPV) of 56%, indicating that a positive initial screening result had a probability of just over one in two of being clinically actionable CAD. Conversely, a negative result provided a reassuring 88% likelihood of CAD absence. Decision curve analysis showed that sequential screening offered optimal clinical benefit for patients with intermediate risk (decision thresholds of 8–38%), balancing diagnostic accuracy, clinical resource utilization and radiation exposure (Fig. 3b). Furthermore, this strategy also showed benefits in the SMuRF-less subgroup, with NNS of 3 versus 7.2 in the indexed-troponin model triaging approach versus universal scanning, higher sensitivity (90%), fewer missed cases (10.4%) and approximately 47% fewer scans, albeit at the cost of lower specificity (53%).

To evaluate whether the multivariable hs-cTnI model might be over-optimistic and to quantify the impact of low-level assay imprecision, we refitted the final model in a fully Bayesian error-in-variables framework. In this approach, the true (latent) log-troponin for each participant is estimated jointly with the regression coefficients, while the assay’s heteroscedastic coefficient of variation (20% below 2.5 ng l^−^^1^; 5% above) is propagated through to all predictions. Across fivefold internal cross-validation, the posterior median AUC was 79.9% (95% credible interval 79.6–80.0%) (Fig. 3c). Decile-wise calibration remained excellent; observed event rates fell in the 95% posterior bands for every risk bin (Fig. 3c). We also performed sensitivity analysis using more conservative and optimistic coefficient of variation (CV) values, which showed stable AUC (0.795–0.80) across these values.

The probability threshold that maximized Youden’s *J* was narrowly distributed (median 0.28, 95% CI 0.23–0.31), indicating that the proposed 30% threshold is robust to both sampling variation and analytical noise at the low troponin range. Pareto-smoothed importance sampling leave-one-out (PSIS-LOO) yielded an expected log-predictive density (elpd_loo) of −763 (s.e. 21) with only two observations showing mildly elevated Pareto-*k* values—which flags influential observations—(0.7 < *k* ≤ 1.0). No very bad (*k* > 1) points were detected, suggesting single outliers do not drive the model’s predictions.

**Fig. 4:**
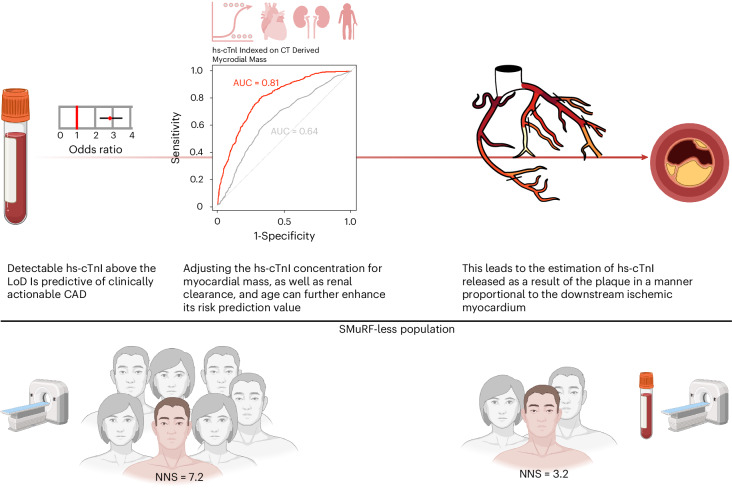
hs-cTnI reflects CT-determined CAD burden in a manner proportional to the downstream myocardial mass supplied by coronary plaque. Adjusting hs-cTnI concentration for myocardial mass, renal clearance and age enhances its predictive value for clinically actionable CAD. Incorporating these adjustments enables improved identification of individuals with significant, yet stable, CAD. The addition of a triaged scanning approach—first risk stratification using the adjusted hs-cTnI model, followed by confirmatory CT CACS—results in a 60% reduction in the need for CT imaging to detect a case of clinically actionable CAD, while maintaining clinically meaningful sensitivity and specificity. Figure created in BioRender; Figtree, G. https://biorender.com/9twui5s (2026).

Collectively, these results demonstrate a clinical framework for a two-stage approach to detect clinically actionable CAD via hs-cTnI-enriched CTCA screening (Fig. 4).

New diagnostic tools and clinical pathways are required for equitable access to screening for CAD in asymptomatic individuals to provide a much-needed new approach to heart attack prevention. Here we demonstrate the potential utility of hs-cTnI in primary prevention settings to help identify patients with clinically actionable CAD on CCTA, particularly those who are otherwise deemed low-risk in the community. In this low-risk group, we show that triaging with hs-cTnI (>2.3 ng l^−1^) increases the chance of CCTA-detected clinically actionable CAD by up to fourfold. The prognostic value of hs-cTn was substantially enhanced by incorporating LVM, age and renal clearance, improving the AUC from 0.64 to 0.81. Although the demonstrated value of LVM adjustment is consistent with the hypothesis that myocardial volume contributes to low-level hs-cTnI release, a simpler hs-cTnI-by-sex model performed similarly to the hs-cTnI-by-LV mass model. This likely relates to the very strong relationship of sex and LVM (noting that when sex was included in LVM in the model, it was not a significant contributor). This has practical implications, suggesting that sex and BMI or BSA could be used to replace LVM in clinical settings where LVM is unavailable. When embedded into a sequential triaging program, the indexed-troponin model improved the efficiency of CT imaging for silent CAD, doubling the diagnostic yield per scan. This resulted in an improved NNS from 3.4 to 1.8 in the total cohort and from 7.2 to 3 in SMuRF-less individuals. This work paves the groundwork for future dedicated prospective trials to validate our findings and examine whether our proposed triaged screening improves patient selection and long-term outcomes.

Although we acknowledge the trajectory toward lower radiation dose CACS, this does not, in itself, imply that universal CT-based screening will become feasible or appropriate at scale. Even at contemporary doses (~0.5 to 1 mSv), population-wide CACS would require substantial expansion in scanner capacity, radiographer workforce and downstream clinical pathways, with inevitable opportunity costs and inequities across resource-limited or geographically remote settings. As highlighted in prior perspectives from our group and others, CAC may ultimately play an expanded role even in apparently low-risk individuals^20^; however, the central implementation challenge is not radiation alone, but scalability, access and health system throughput. In that context, a blood-based triage step retains practical and clinical value: in our data, an hs-cTnI-based sequential strategy reduced CT examinations by ~59–60% while maintaining an NPV of ~87% for excluding clinically actionable CAD, thereby enriching imaging yield and potentially enabling more equitable deployment of CT resources. We therefore view hs-cTnI not as a competitor to CACS, but as a complementary upstream filter that can help operationalize targeted imaging strategies—particularly in SMuRF-less and otherwise ‘low-risk’ groups in which current primary prevention algorithms underperform.

It is worth highlighting that a normal hs-cTnI in this framework should not be interpreted as excluding clinically actionable CAD. Rather, hs-cTnI is better conceptualized as an upstream enrichment tool to improve the allocation of downstream CT resources. Individuals with a low hs-cTnI-based score would still warrant ongoing guideline-based preventive care and could undergo repeat biomarker assessment or coronary imaging if their overall risk profile, family history, symptom trajectory or other clinical features remained concerning. Thus, the proposed role of hs-cTnI is one of risk down-classification, not absolute rule-out. Furthermore, the balance between missed cases and scan reduction is inherently threshold-dependent, and future prospective implementation studies should determine the threshold that best matches the intended clinical objective, with more sensitivity-favoring thresholds likely appropriate when minimizing missed disease is prioritized.

The Bayesian reanalysis supports the internal validity and clinical robustness of the hs-cTnI-triaging strategy: the discriminative performance is stable after accounting for measurement error, the calibration curve is well aligned with observed outcomes, and the chosen rule-in threshold remains well defined. These findings support the use of the indexed-troponin score to triage targeted coronary imaging, aiming for early, personalized prevention of atherosclerotic CAD.

Although symptom status is central to current diagnostic pathways, it is an imperfect surrogate for underlying coronary atherosclerotic burden. Contemporary evidence has shown that traditional age–sex–symptom pre-test probability models substantially overestimate obstructive CAD in modern populations undergoing CT evaluation, and recent guidelines now favor risk factor-weighted clinical likelihood rather than symptom category alone^21^. In parallel, a substantial proportion of patients presenting with angina symptoms do not have obstructive epicardial CAD, reflecting alternative mechanisms such as microvascular or vasospastic disease, or noncardiac origins. In this context, an objective blood-based biomarker may provide complementary value by refining triage when symptoms are absent, atypical or of uncertain significance.

Our study demonstrates the utility of hs-cTn, adjusted for LVM, as a potential screening tool to identify individuals at increased risk of significant CAD in a nonacute adult population with suspected chronic coronary syndrome, using an approach that is particularly effective in those without traditional risk factors. This builds on previously published data that have provided strong evidence for the association of hs-cTn with an increased risk of CVD and CAD-related events^16^. However, the use of hs-cTn to triage to CT imaging for early detection of CAD before a heart attack provides the advantage and opportunity for effective intervention to achieve an effective remission. This is seen in large-scale secondary prevention studies where intensive LDL lowering (< 1.4 mM) results in plaque stabilization or even regression^22,23^ and decreases the risk of major adverse cardiovascular events in large-scale clinical outcome studies^24^. Furthermore, mortality benefits are observed in individuals who have developed their CAD despite a low LDL concentration^25^.

An important methodological question is whether the effect of LVM adjustment simply reflects sex-related differences in myocardial size. In our analysis, however, the incremental improvement associated with the hs-cTnI × LVM interaction was evident before sex was introduced into the subsequent model, and the optimal unadjusted hs-cTnI thresholds were very similar in men and women. Together, these findings suggest that myocardial mass is not acting solely as a proxy for sex, but may capture biologically relevant variation in the amount of downstream myocardium contributing to circulating troponin.

In the current study, hs-cTnI was strongly correlated with semi-quantitative measures of CAD burden, including CACS, Gensini and noncalcified plaque score. The validity of this finding is supported by the demonstration of a potential pathophysiological mechanism in this nonacute cohort. The stronger association of hs-cTnI with absolute CACS, compared with CACS percentile (adjusted for age and sex), is consistent with the marker levels being a consequence (or downstream) of plaque likely via myocardial micro-injury^26^ as opposed to being upstream and reflective of mechanisms involved in an individual’s susceptibility to CAD^27^. The likely role of myocardial microinjury to explain the mechanism of hs-cTnI with plaque burden is further supported by the striking role of lesion location in the strength of the relationship, being stronger the more myocardium supplied downstream of the plaque. These data are consistent with a previous study that suggested that hs-cTnI release is a result of platelet adherence and activation to regions of atherosclerotic plaque, with distal microembolization and myocardial injury^28^.

An optimal cutoff of 2.20–2.50 ng l^−1^ for hs-cTnI was found to have the best classification for clinically actionable CAD, resulting in an AUC of 0.62 in the full cohort and 0.68 in the SMuRF-less group. This cutoff is distinct from standard approaches using the 99th percentile Upper Reference Limit as the marker, which for the Abbott hs-cTnI assay is defined as 28 ng l^−1^ (refs. ^29,30^). This cutoff is close to the LoD and may have higher variation at lower concentrations, which is a limitation of current high-sensitivity assays. Recent studies, however, have demonstrated that thresholds lower than the 99th and close to the LoD can have prognostic value, both in acute and chronic coronary syndrome populations. A joint research effort involving ~4,500 patients to support the National Institute for Health and Care Excellence guidelines for the use of troponin as a safe rule-out for chest pain patients in the emergency department showed that applying a limit of 2.00 ng l^−1^ for hs-cTnI in patients presenting with a thrombolysis in myocardial infarction (TIMI) score of 0 achieved sensitivity in the range of 98.9–99.5% and an NPV of 99.5% for major adverse cardiac event at 30 days^31^. Furthermore, the use of a low-risk electrocardiogram combined with hs-cTnI ≤3 in a cohort comprising 1,040 patients exhibited an impeccable 100% sensitivity in excluding instances of cardiac rehospitalization or percutaneous coronary intervention over a 6-month span^32^. Thus, although the derived cutoff in our cohort is close to the LoD, its prognostic ability appears consistent with other recent high-quality studies, at least those testing a ‘rule-out’ utility.

Our results show that hs-cTnI can enrich apparently low-risk nonacute populations for the presence of clinically actionable CAD, known to be associated with a high rate of MI^33^, and drive intensive medical treatment as per new guidelines^34,35^. This may be particularly valuable in those who develop CAD despite low traditional risk factor burden (SMuRF-less)^4^ who do not meet criteria for preventative therapy or screening CT scan. Although the use of hs-cTnI is not diagnostic in this stable population, using levels above 2.3 ng l^−1^ to guide screening CT decisions has the potential to improve the yield of this powerful and accurate imaging tool, with implications for scale and equity. Data presented in this study support the value of a prospective study, using hs-cTnI ≥2.3 to triage individuals for CT. Similar to the study design we implemented for CAD polygenic risk scoring in the ESCALATE Study^36^ (recruitment closed, analysis in progress), the prognostic power of hs-cTnI could be prospectively tested in primary care to see whether it can reduce the number of CT scans needed to detect patients with clinically actionable CAD, and lead to appropriate intensive medical treatment in those shown to have the disease. Longer term, larger studies could consider the impact of implementing this hs-cTnI–CT decision pathway on plaque progression and ultimately heart attack prevention.

The strength of our study lies in the size of the cohort and its characterization using CCTA, which takes into account the burden and characteristics of plaque, as well as the location and downstream myocardium supplied by the plaque. Our integrated methodology, which integrates confounding factors to enhance the predictive power of hs-cTnI, significantly improves the detection of subclinical CAD in individuals remote from an ACS. The following limitations of this study should be considered. First, our cohort has a high proportion of white participants and may not be generalizable to populations with higher ethnic diversity. Second, the study cohort was not a pure primary prevention population, but a pragmatic real-world mixture of patients referred with stable symptoms suspicious for CAD or asymptomatic individuals referred for screening. Although this limits the extent to which the full cohort alone can define the role of hs-cTnI specifically as a screening biomarker, it also reflects the reality that symptom-based referral pathways are frequently heterogeneous and clinically uncertain. Strong performance demonstrated by the sensitivity analysis in the asymptomatic subgroup, provides the most direct support for a potential screening application of hs-cTnI, whereas the combined cohort informs potential pragmatic utility in contemporary referral practice. Third, our study endpoint of ‘clinically actionable’ CAD is based on CT detection of atherosclerosis above a threshold for treatment according to clinical guidelines. The BioHEART study is not yet powered for validation against clinical outcomes such as MI. Fourth, our study is retrospective, and prospective testing is warranted. We would advocate for a rapid translational implementation study akin to the ESCALATE study to evaluate whether the LVM-adjusted hs-cTnI could improve the detection of subclinical CAD in primary care^36^. If positive, testing the clinical utility of hs-cTnI-based enrichment for CTCA to impact treatment and reduce MI would be warranted. Fifth, the pragmatic use of hs-cTnI requires adapting the reporting methods currently used in the clinical setting. Our proposed cutoffs, although higher than the LoD of the Abbott hs-cTnI assay, are lower than the standard lower limit of quantitation (3.20 ng l^−1^) based on a coefficient of variation limit of 20%^37,38^. Aside from the practical implications of reporting these results, the increased imprecision at low-concentration decision points raises the risk of misclassification^39^. The performance of hs-cTnI analysis in replicates could mitigate this risk in clinical practice, particularly if performed serially. Moreover, recent investigations underscore the necessity of considering macrotroponin as a potential cause of persistent troponin elevation, particularly in the COVID-19 era, to prevent unnecessary diagnostic procedures and clinical anxiety^40^. Finally, the false-negative proportion observed in the current retrospective modeling indicates that hs-cTnI should not be used in isolation to exclude CAD, but rather as one component of a broader, longitudinal risk-stratification strategy and a decision aid regarding the use of CT imaging.

## Discussion

We demonstrate that hs-cTn can be utilized as a noninvasive, inexpensive, and readily available biomarker with the potential to enrich the asymptomatic population for the likelihood of subclinical CAD being identified at CCTA. Screening, particularly in SMuRF-less patients or otherwise low-risk individuals, with this hs-cTnI-triage to CCTA clinical pathway(with or without normalization for LV mass) may be a cost-effective strategy to help address the recent call by the Lancet Commission for Rethinking CAD, and particularly the need for diagnostic pathways to help pick up CAD before an acute event. Providing equitable access to clinical pathways for detecting and treating chronic CAD before a heart attack has the potential to reduce cardiovascular morbidity and mortality dramatically. Future prospective implementation studies are warranted to test whether hs-cTnI-guided triage meaningfully reduces downstream imaging requirements and long-term cardiovascular events, particularly among SMuRF-less individuals and younger women, where early detection is most challenging yet clinically important.

## Methods

BioHEART-CT is a multicenter, longitudinal, prospective, observational cohort study of people with outpatient referrals for CCTA to investigate suspected CAD in symptomatic patients or for screening in otherwise asymptomatic patients (~50%). The study design has been described in detail previously^41^. Informed consent was obtained at the time of enrollment, and participants who withdrew their consent were excluded from this analysis. The study has been approved by the Northern Sydney Local Health District Human Research Ethics Committee (2019/ETH08376) and registered on the Australian and New Zealand Clinical Trial Registry (ACTRN12618001322224).

### Study population

**Extended Data Fig. 1:**
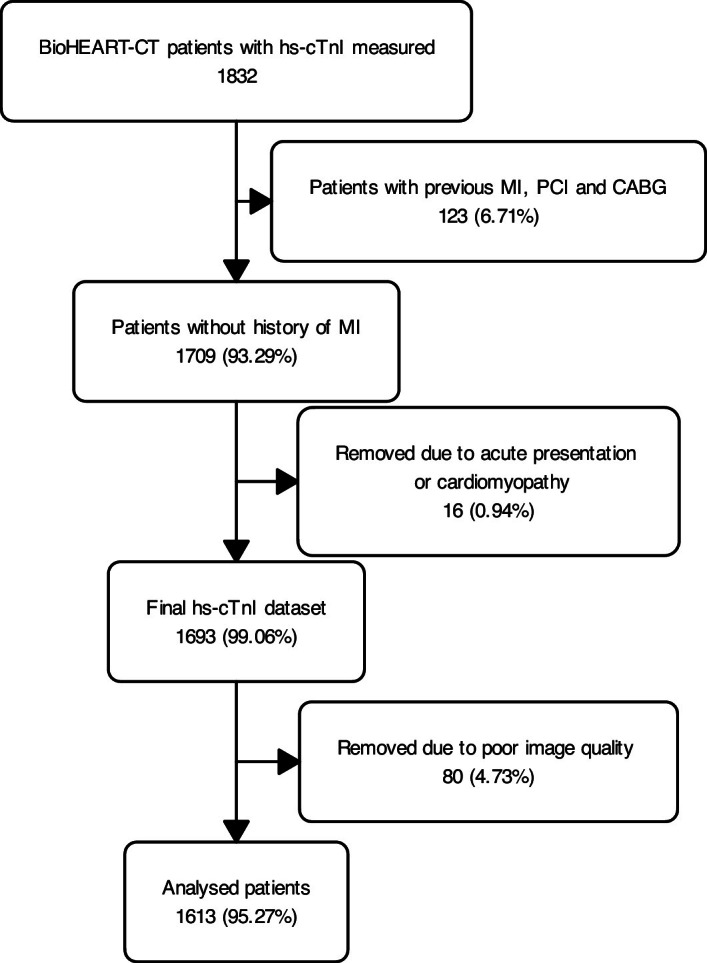
Patient inclusion flowchart.

**Extended Data Fig. 2:**
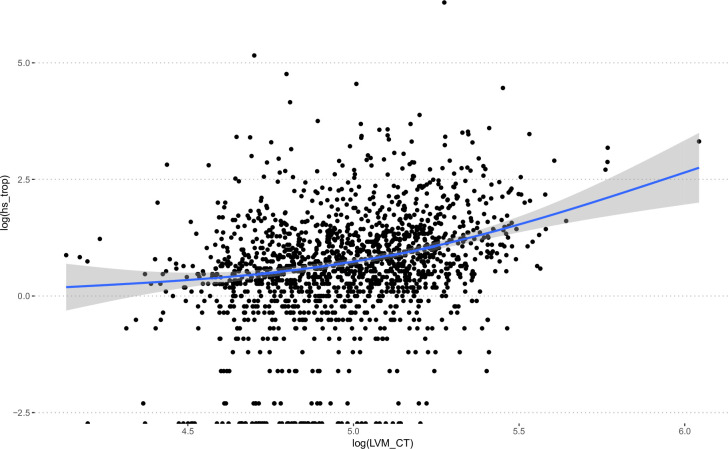
Association of log-transformed LV mass and hs-cTnI. Outcome-stratified bootstrap resampling (CACS ≥100 vs <100) was performed to preserve class balance. Within each bootstrap resample, the hs-cTnI cutpoint maximising Youden’s J (sensitivity + specificity − 1) was re-estimated. Top: density of the resulting optimal cutpoints across resamples (rug marks indicate individual resamples). Bottom: distribution of out-of-bag Youden’s J values across resamples.

**Extended Data Fig. 3:**
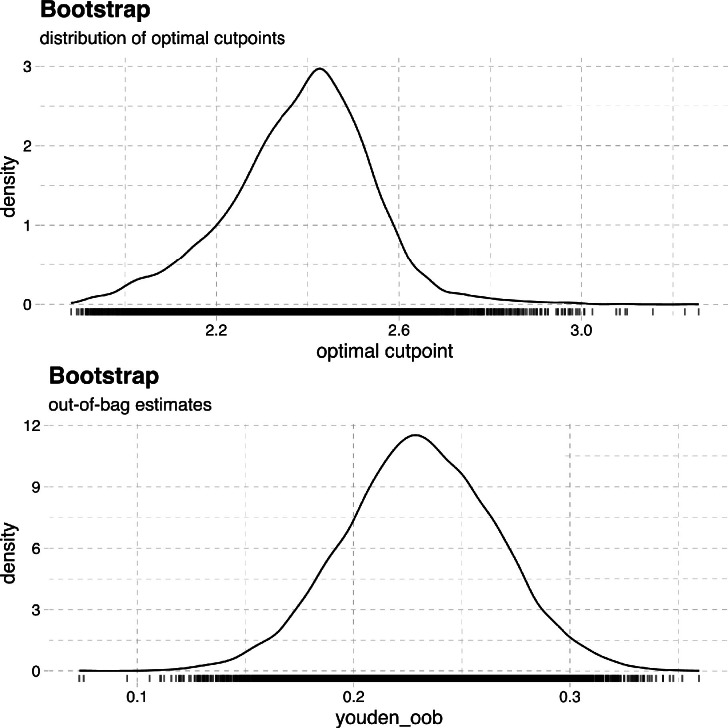
Outcome-stratified bootstrap optimisation of hs-cTnI cutpoint for prediction of CACS ≥100.

**Extended Data Fig. 4:**
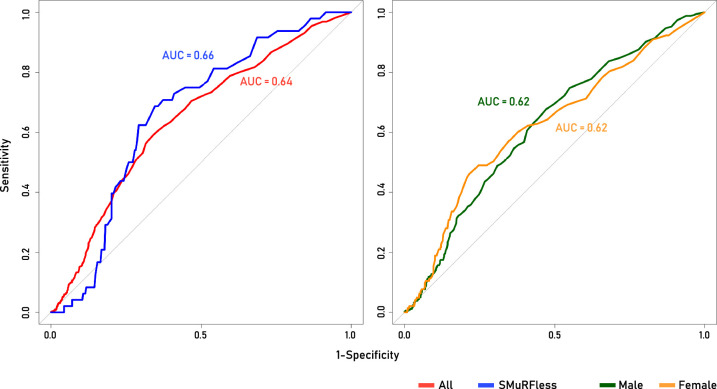
ROC-AUC for hs-cTnI in predicting clinically actionable CAD. **a**) the overall group and the SMuRF-less subgroup, **b**) Male vs female subgroups.

The current paper is a retrospective analysis of 1,613 adult participants from the BioHEART-CT study with measured hs-cTnI, enrolled between 2015 and 2021 who met the following inclusion criteria: (1) able to provide informed consent; (2) no prior documented history of cardiomyopathy; (3) MI—defined as previous history of ACS—or coronary revascularization (comprising of percutaneous coronary intervention or coronary artery bypass surgery); (4) adequate blood sample available; and (5) CCTA imaging studies passing quality control (Extended Data Fig. 1).

### Variables and definitions

Baseline data were collected through structured interviews during enrollment, including demographics, anthropometrics, medical history, medications, occupational and environmental exposures, and recent cardiac symptoms. Current smoking was defined as having smoked tobacco products in the past 12 months. Significant smoking was defined as a 10-pack-year smoking history. Significant family history was defined as a first-degree relative experiencing ischemic heart disease under 60 years of age.

The number of SMuRFs was defined by self-report: hypertension, hypercholesterolemia, diabetes and significant smoking. SMuRF-less individuals refer to all those with zero reports of SMuRFs.

### Coronary CT angiography

CCTA scans were performed using standard clinical protocols, and radiation doses were minimized per current recommendations^42^. Coronary artery reconstructions were performed using vendor software.

### CCTA disease severity scores

CAD burden was assessed using the CACS as described in the Agatston method^43^ (hyperattunated areas of at least 1 mm^2^ or three or more adjacent pixels with >130 Hounsfield units). Clinically actionable CAD was defined as CACS ≥ 100 consistent with guideline-endorsed thresholds where intensification of lipid-lowering therapy and, in selected patients, aspirin are recommended^44^. Sensitivity analyses were also performed for CACS > 0 and CACS percentile ≥75th to capture milder atherosclerosis and age-adjusted burden. Quantitative measures of total plaque burden (including noncalcified and calcified plaque) were available for the Discovery cohort (*n* = 1,000), which included a 17-segment Gensini stenosis score. By incorporating a plaque composition multiplier for each scored segment, we were able to quantify the noncalcified plaque score^45^.

### Myocardial mass estimation

To estimate myocardial mass on CCTA, we automated heart chamber segmentation using the TotalSegmentator^46^ module, using nnUNet-based deep learning^47^ in Python through our custom data management and processing pipeline. As a quality control measure, a trained physician visually validated a randomly selected subset of 100 segmentations using the 3D Slicer imaging platform^48^. LVM was then calculated using the following formula: $${\mathrm{{LVM}}}=1.05\frac{\mathrm{g}}{\mathrm{ml}}$$$$(\mathrm{specific}\mathrm{gravity}\mathrm{of}\mathrm{myocardial}\mathrm{tissue})\times \mathrm{LV}\mathrm{volume}$$. Further validation was performed using TTE-derived LV wall thickness. TTE results were only available for 359 patients, whereas CCTA-derived LVM was available for all but 59 patients.

### Hs-cTnI assay

Hs-cTnI concentration was measured on the same day as the CCTA, either just before or after the scan, in thawed EDTA plasma aliquots using Abbott ARCHITECT *i*1000 and the Beckman Access 2 analyzers in the Royal Prince Alfred Hospital Department of Pathology in Sydney, Australia. The LoD for this assay is 0.1 ng l^−1^.

### Statistical analysis

All statistical analyses were performed with R (v.4.1.1) in RStudio. Continuous variables were summarized as mean ± s.d. or median [interquartile range (IQR)], according to distributional normality assessed by quantile–quantile plots and the Shapiro–Wilk test; right-skewed variables were log_2_-transformed. Spearman’s rank correlation coefficients (*ρ*) quantified associations between hs-cTnI and clinical or CCTA measures.

An optimal hs-cTnI threshold for clinically actionable CAD (CACS ≥ 100) was derived by maximizing Youden’s index over 10,000 stratified bootstrap resamples with the threshold re-estimated independently in each resample to account for sampling variability and stabilize the estimate the reported threshold is the median across resamples, with percentile-based uncertainty bounds. Univariable logistic regression identified candidate predictors (*P* < 0.05) for entry into multivariable models. We then constructed sequential logistic regression models to assess the incremental discrimination conferred by:

1. Model 1: hs-cTnI dichotomized at the optimal threshold.
2. Model 2: Model 1 + CT-derived myocardial mass (interaction term hs-cTnI × LVM).
3. Model 3: Model 2 + renal function (estimated glomerular filtration rate), sex and prespecified interaction terms to account for the effect of age on all variables (both on the dependent variable, risk of CAD and independent variables) and the interaction between sex and LVM.

Model discrimination was evaluated by AUC, and AUC comparisons used DeLong’s test. Multicollinearity was assessed by variance inflation factors <2. Decision curve analysis was conducted to estimate the net clinical benefit across threshold probabilities ranging from 8% to 38%. Bayesian post-test probabilities (PPV and NPV) were computed using observed cohort prevalence. To compare screening strategies—universal CACS for all versus a two-stage sequential approach—metrics calculated included sensitivity, specificity, PPV and NPV, diagnostic yield (cases per scan), number needed to screen $\left({\mathrm{NNS}}={\frac{1}{{\mathrm{Diagnostic}}\;{\mathrm{yield}}}}\right)$ and percentage reduction in scans. Statistical significance was defined as two-tailed *P* < 0.05.

To ensure robust internal validation and minimize overfitting, we used multiple resampling strategies. First, a stratified fivefold cross-validation framework was implemented, with each held-out fold predicted by a model trained on the remaining data. Second, the optimal hs-cTnI threshold was stabilized using 10,000 bootstrapped resamples, maximizing Youden’s index. Third, a Bayesian error-in-variables (EIV) model explicitly propagated assay imprecision at low concentrations and re-estimated discrimination on held-out folds. Together, these complementary approaches quantified out-of-sample discrimination, calibration and robustness to analytical noise, the core aims of derivation and validation. A conventional 70/30 derivation-validation split would have reduced statistical power for model training and limited subgroup representation (for example, SMuRF-less individuals and younger women), both of which were key populations of interest. For this reason, we opted for repeated resampling and cross-validation—well-accepted internal validation strategies in biomarker development studies. Findings are presented as hypothesis-generating and warranting prospective implementation.

### Bayesian uncertainty modeling and internal validation

Replicate measurements were not performed in the BioHEART-CT cohort, because hs-cTnI was analyzed once per participant following standard clinical laboratory protocols to minimize sample volume requirements and align with real-world assay use. Instead, we relied on external validation data from manufacturer precision studies and peer-reviewed literature. To address potential overfitting and analytical imprecision at very low hs-cTnI concentrations, we developed a Bayesian EIV logistic regression model. The model treats the true (latent) log-transformed hs-cTnI value for each participant as an unobserved parameter and explicitly propagates assay measurement error:

1. Measurement error. Below 2.5 ng l^−1^, we assumed a 20% CV; above this threshold, the CV was set to 5%. Observed hs-cTnI was modeled as Normal (true value, s.d. determined by the appropriate CV). For sensitivity analysis, we also considered more conservative (reflecting potential interlaboratory variability or sample matrix effects; CV 30% and 7.5%) and optimistic (aligning with in-run precision at optimal conditions; CV 10% and 2.5%) scenarios.
2. Linear predictor. Interaction terms were retained exactly as in Model 3 (hs-cTnI × LVM and hs-cTnI × creatinine, with age interactions), allowing the Bayesian analysis to evaluate the same clinical hypothesis while quantifying parameter uncertainty.
3. Priors. Weakly informative normal priors centered on the maximum-likelihood estimates (*σ* = 1 for main effects, *σ* = 0.5 for interaction terms) restrained extreme solutions without overwhelming the data.
4. Sampling. The model was fitted using four chains, with 4,000 post-warm-up draws per chain, and three in-chain threads (resulting in a total of 12 Apple-silicon cores). adapt_delta was set to 0.98 to avoid divergent transitions; no divergences were observed.
5. Internal validation. We performed stratified fivefold cross-validation, refitting the model on 80% of the data and evaluating posterior predictions on the held-out fold. For each fold, we drew 1,000 posterior samples of the predicted probability and calculated AUC. In addition, PSIS-LOO was computed on the full model to identify influential observations. PSIS-LOO is a cross-validation approximation for assessing out-of-sample predictive performance
6. Quantifying analytic fragility. Posterior samples of the ‘optimal’ probability cutoff (maximizing Youden’s *J*) were extracted for every draw, giving a distribution of clinically actionable thresholds rather than a single point estimate.

### Reporting summary

Further information on research design is available in the Nature Portfolio Reporting Summary linked to this article.

## Supplementary information

Reporting Summary

Peer Review File

Supplementary Tables 1–3

## Data Availability

The data supporting this study’s findings are not openly available for reasons of sensitivity and ethical consideration. Collaborative research proposals can be submitted for consideration under the BioHEART Presentations, Publications, and Data Access policy. Data will be provided as approved by ethics and governance following internal review. Individuals interested in learning more about this opportunity are encouraged to contact Professor Gemma A. Figtree at Gemma.Figtree@sydney.edu.au.

## References

[1] Vernon, S. T. et al. ST-segment-elevation myocardial infarction (STEMI) patients without standard modifiable cardiovascular risk factors—how common are they, and what are their outcomes? *J. Am. Heart Assoc.* **8**, e013296 (2019). 31672080 10.1161/JAHA.119.013296 PMC6898813

[2] Vernon, S. T. et al. Increasing proportion of ST elevation myocardial infarction patients with coronary atherosclerosis poorly explained by standard modifiable risk factors. *Eur. J. Preven. Cardiol.* **24**, 1824–1830 (2017). 10.1177/2047487317720287 28703626

[3] Figtree, G. A. et al. Mortality in STEMI patients without standard modifiable risk factors: a sex-disaggregated analysis of SWEDEHEART registry data. *Lancet* **397**, 1085–1094 (2021). 33711294 10.1016/S0140-6736(21)00272-5

[4] Figtree, G. A. & Vernon, S. T. Coronary artery disease patients without standard modifiable risk factors (SMuRFs) — a forgotten group calling out for new discoveries. *Cardiovasc. Res.* **117**, e76–e78 (2021). 34037711 10.1093/cvr/cvab145

[5] Ahmadi, A., Argulian, E., Leipsic, J., Newby, D. E. & Narula, J. From subclinical atherosclerosis to plaque progression and acute coronary events. *J. Am. Coll. Cardiol.* **74**, 1608–1617 (2019). 31537271 10.1016/j.jacc.2019.08.012

[6] Kataoka, Y., Andrews, J., Puri, R., Psaltis, P. J. & Nicholls, S. J. Plaque burden, microstructures and compositions underachieving very low LDL-C levels. *Curr. Opin. Endocrinol. Diabetes Obes.* **24**, 122–132 (2017). 28107247 10.1097/MED.0000000000000317

[7] Di Giovanni, G., Kataoka, Y., Bubb, K., Nelson, A. J. & Nicholls, S. J. Impact of lipid lowering on coronary atherosclerosis moving from the lumen to the artery wall. *Atherosclerosis* **367**, 8–14 (2023). 36716526 10.1016/j.atherosclerosis.2023.01.017

[8] Zanda, G. & Varbella, F. Stabilization of vulnerable plaque in the ACS patient: evidence from HUYGENS studies. *Eur. Heart J. Suppl.* **25**, C106–C108 (2023). 37125301 10.1093/eurheartjsupp/suad013 PMC10132721

[9] Navarese, E. P. et al. Association between baseline LDL-C level and total and cardiovascular mortality after LDL-C lowering: a systematic review and meta-analysis. *JAMA* **319**, 1566–1579 (2018). 29677301 10.1001/jama.2018.2525 PMC5933331

[10] Figtree, G. A. A Quest for Zero Heart Attacks. *Scientia* https://www.scientia.global/professor-gemma-figtree-a-quest-for-zero-heart-attacks/ (2023).

[11] Bergström, G. et al. Prevalence of subclinical coronary artery atherosclerosis in the general population. *Circulation* **144**, 916–929 (2021). 34543072 10.1161/CIRCULATIONAHA.121.055340 PMC8448414

[12] Zaman, S. et al. The Lancet Commission on rethinking coronary artery disease: moving from ischaemia to atheroma. *Lancet* **405**, 1264–1312 (2025). 40179933 10.1016/S0140-6736(25)00055-8 PMC12315672

[13] Welsh, P. et al. Cardiac troponin T and troponin I in the general population. *Circulation* **139**, 2754–2764 (2019). 31014085 10.1161/CIRCULATIONAHA.118.038529 PMC6571179

[14] Caselli, C. et al. Effect of coronary atherosclerosis and myocardial ischemia on plasma levels of high-sensitivity troponin T and NT-proBNP in patients with stable angina. *Arter. Thromb. Vasc. Biol.* **36**, 757–764 (2016). 10.1161/ATVBAHA.115.306818 26868212

[15] Gaiki, M. R., DeVita, M. V., Michelis, M. F., Panagopoulos, G. & Rosenstock, J. L. Troponin I as a prognostic marker of cardiac events in asymptomatic hemodialysis patients using a sensitive troponin I assay. *Int. Urol. Nephrol.* **44**, 1841–1845 (2012). 22311387 10.1007/s11255-012-0128-x

[16] Shah, A. S. V. et al. Cardiac troponins and cardiovascular disease risk prediction. *J. Am. Coll. Cardiol.* **85**, 1471–1484 (2025). 40204376 10.1016/j.jacc.2025.02.016 PMC13266502

[17] Global Heart Hub. *Achieving Early Detection and Diagnosis of Cardiovascular Disease: A Manifesto for Change* (Global Heart Hub, 2025). 10.1093/eurheartj/ehae455 39656480

[18] Fathieh, S. et al. Evaluating the role of lipoprotein(a) in enhancing risk stratification for the presence and extent of subclinical coronary artery disease burden: a BioHEART-CT study. *Eur. J. Prevent. Cardiol.*10.1093/eurjpc/zwaf323 (2025). 10.1093/eurjpc/zwaf323 40458002

[19] Figtree, G. A. et al. Clinical pathway for coronary atherosclerosis in patients without conventional modifiable risk factors: JACC state-of-the-art review. *J. Am. Coll. Cardiol.* **82**, 1343–1359 (2023). 37730292 10.1016/j.jacc.2023.06.045 PMC10522922

[20] Playford, D., Hamilton-Craig, C., Dwivedi, G. & Figtree, G. Examining the potential for coronary artery calcium (CAC) scoring for individuals at low cardiovascular risk. *Heart Lung Circ.* **30**, 1819–1828 (2021). 34332891 10.1016/j.hlc.2021.04.026

[21] Vrints, C. et al. 2024 ESC Guidelines for the management of chronic coronary syndromes: Developed by the task force for the management of chronic coronary syndromes of the European Society of Cardiology (ESC) Endorsed by the European Association for Cardio-Thoracic Surgery (EACTS). *Eur. Heart J.* **45**, 3415–3537 (2024). 39210710 10.1093/eurheartj/ehae177

[22] Nicholls, S. J. et al. Effect of evolocumab on coronary plaque phenotype and burden in statin-treated patients following myocardial infarction. *JACC Cardiovasc. Imaging* **15**, 1308–1321 (2022). 35431172 10.1016/j.jcmg.2022.03.002

[23] Nicholls, S. J. et al. Intravascular ultrasound-derived measures of coronary atherosclerotic plaque burden and clinical outcome. *J. Am. Coll. Cardiol.* **55**, 2399–2407 (2010). 20488313 10.1016/j.jacc.2010.02.026

[24] Jun, M. et al. Effects of fibrates on cardiovascular outcomes: a systematic review and meta-analysis. *Lancet* **375**, 1875–1884 (2010). 20462635 10.1016/S0140-6736(10)60656-3

[25] Baigent, C. et al. Efficacy and safety of more intensive lowering of LDL cholesterol: a meta-analysis of data from 170,000 participants in 26 randomised trials. *Lancet* **376**, 1670–1681 (2010). 21067804 10.1016/S0140-6736(10)61350-5 PMC2988224

[26] Kop, W. J. et al. Cardiac microinjury measured by troponin T predicts collagen metabolism in adults aged ≥65 years with heart failure. *Circ. Heart Fail.* **5**, 406–413 (2012). 22685114 10.1161/CIRCHEARTFAILURE.111.965327 PMC4479498

[27] Januzzi, J. L. et al. High-sensitivity troponin I and coronary computed tomography in symptomatic outpatients with suspected CAD: insights from the PROMISE trial. *JACC Cardiovasc. Imaging* **12**, 1047–1055 (2019). 29550314 10.1016/j.jcmg.2018.01.021 PMC6129434

[28] Freda, B. J., Tang, W. W., Van Lente, F., Peacock, W. F. & Francis, G. S. Cardiac troponins in renal insufficiency: review and clinical implications. *J. Am. Coll. Cardiol.* **40**, 2065–2071 (2002). 12505215 10.1016/s0735-1097(02)02608-6

[29] Wu, A. H. B. et al. Clinical laboratory practice recommendations for the use of cardiac troponin in acute coronary syndrome: expert opinion from the Academy of the American Association for Clinical Chemistry and the Task Force on Clinical Applications of Cardiac Bio-Markers of the International Federation of Clinical Chemistry and Laboratory Medicine. *Clin. Chem.* **64**, 645–655 (2018). 29343532 10.1373/clinchem.2017.277186

[30] Thygesen, K. et al. Fourth universal definition of myocardial infarction (2018). *Circulation* **138**, e618–e651 (2018). 30571511 10.1161/CIR.0000000000000617

[31] Carlton, E. W. et al. Assessment of the 2016 National Institute for Health and Care Excellence high-sensitivity troponin rule-out strategy. *Heart* **104**, 665–672 (2018). 28864718 10.1136/heartjnl-2017-311983 PMC5890641

[32] Neumann, J. T. et al. Immediate rule-out of acute myocardial infarction using electrocardiogram and baseline high-sensitivity troponin I. *Clin. Chem.* **63**, 394–402 (2017). 27903616 10.1373/clinchem.2016.262659

[33] Budoff, M. J. et al. Ten-year association of coronary artery calcium with atherosclerotic cardiovascular disease (ASCVD) events: the multi-ethnic study of atherosclerosis (MESA). *Eur. Heart J.* **39**, 2401–2408 (2018). 29688297 10.1093/eurheartj/ehy217 PMC6030975

[34] Visseren, F. L. J. et al. 2021 ESC Guidelines on cardiovascular disease prevention in clinical practice. *Eur. Heart J.* **42**, 3227–3337 (2021). 34458905 10.1093/eurheartj/ehab484

[35] Blumenthal, R. S. et al. 2026 ACC/AHA/AACVPR/ABC/ACPM/ADA/AGS/APhA/ASPC/NLA/PCNA guideline on the management of dyslipidemia: a report of the American College of Cardiology/American Heart Association Joint Committee on Clinical Practice Guidelines. *Circulation* **153**, e1154–e1276 (2026). 41824552 10.1161/CIR.0000000000001423

[36] Gray, M. P. et al. Incorporating a polygenic risk score-triaged coronary calcium score into cardiovascular disease examinations to identify subclinical coronary artery disease (ESCALATE): protocol for a prospective, nonrandomized implementation trial. *Am. Heart J.* **264**, 163–173 (2023). 37364748 10.1016/j.ahj.2023.06.009

[37] Masotti, S. et al. Evaluation and comparison with other high-sensitivity methods of analytical performance and measured values of a new laboratory test for cardiac troponin I assay. *J. Appl. Lab. Med.* **6**, 1237–1250 (2021). 33829255 10.1093/jalm/jfab017

[38] Wang, J. et al. Evaluation of a newly developed chemiluminescence immunoassay for detecting cardiac troponin T. *J. Clin. Lab. Anal.* **32**, e22311 (2018). 28940712 10.1002/jcla.22311 PMC5873270

[39] Aakre, K. M., Apple, F. S., Mills, N. L., Meex, S. J. R. & Collinson, P. O. Biomarkers tIFoCCCoCAoC. Lower limits for reporting high-sensitivity cardiac troponin assays and impact of analytical performance on patient misclassification. *Clin. Chem.* **70**, 497–505 (2023). 10.1093/clinchem/hvad185 38102065

[40] Kempton, H., Jones, G., McCready, M. & Kovacic, J. Macrotroponin in the COVID-19 era: an under-recognised cause of persistent troponin elevation. *Heart Lung Circ.* **33**, 1147–1150 (2024). 38705780 10.1016/j.hlc.2024.03.007

[41] Kott, K. A. et al. Biobanking for discovery of novel cardiovascular biomarkers using imaging-quantified disease burden: protocol for the longitudinal, prospective, BioHEART-CT cohort study. *BMJ Open* **9**, e028649 (2019). 31537558 10.1136/bmjopen-2018-028649 PMC6756427

[42] Abbara, S. et al. SCCT guidelines for the performance and acquisition of coronary computed tomographic angiography: A report of the Society of Cardiovascular Computed Tomography Guidelines Committee: endorsed by the North American Society for Cardiovascular Imaging (NASCI). *J. Cardiovasc. Comput. Tomogr.* **10**, 435–449 (2016). 27780758 10.1016/j.jcct.2016.10.002

[43] Agatston, A. S. et al. Quantification of coronary artery calcium using ultrafast computed tomography. *J. Am. Coll. Cardiol.* **15**, 827–832 (1990). 2407762 10.1016/0735-1097(90)90282-t

[44] Watts, G. F. et al. Integrated guidance for enhancing the care of familial hypercholesterolaemia in Australia. *Heart Lung Circ.* **30**, 324–349 (2021). 33309206 10.1016/j.hlc.2020.09.943

[45] Gensini, G. G. A more meaningful scoring system for determining the severity of coronary heart disease. *Am. J. Cardiol.* **51**, 606 (1983). 6823874 10.1016/s0002-9149(83)80105-2

[46] Wasserthal, J. et al. TotalSegmentator: robust segmentation of 104 anatomical structures in CT images. *Radiol. Artif. Intell.* **5**, e230024 (2023). 37795137 10.1148/ryai.230024 PMC10546353

[47] Isensee, F., Jaeger, P. F., Kohl, S. A. A., Petersen, J. & Maier-Hein, K. H. nnU-Net: a self-configuring method for deep learning-based biomedical image segmentation. *Nat. Methods* **18**, 203–211 (2021). 33288961 10.1038/s41592-020-01008-z

[48] Fedorov, A. et al. 3D Slicer as an image computing platform for the Quantitative Imaging Network. *Magn. Reson. Imaging* **30**, 1323–1341 (2012). 22770690 10.1016/j.mri.2012.05.001 PMC3466397

