# Supplementary material for: Utility of high-sensitivity cardiac troponin I normalized to left ventricular mass in detection of stable coronary artery disease
Source: Nat Cardiovasc Res. 2026 Jul 30;5(8):763–76. doi: 10.1038/s44161-026-00837-z (PMC13461367; doi:10.1038/s44161-026-00837-z)
Supplement: Supplementary file 1 [file 44161_2026_837_MOESM1_ESM.pdf]

nature portfolio

Corresponding author(s): Gemma A Figtree

Last updated by author(s): May 2, 2026

Reporting Summary

Nature Portfolio wishes to improve the reproducibility of the work that we publish. This form provides structure for consistency and transparency in reporting. For further information on Nature Portfolio policies, see our Editorial Policies and the Editorial Policy Checklist.

Statistics

For all statistical analyses, confirm that the following items are present in the figure legend, table legend, main text, or Methods section.

n/a	Confirmed	
☐	☒	The exact sample size (n) for each experimental group/condition, given as a discrete number and unit of measurement
☐	☒	A statement on whether measurements were taken from distinct samples or whether the same sample was measured repeatedly
☐	☒	The statistical test(s) used AND whether they are one- or two-sided<br>*Only common tests should be described solely by name; describe more complex techniques in the Methods section.*
☐	☒	A description of all covariates tested
☐	☒	A description of any assumptions or corrections, such as tests of normality and adjustment for multiple comparisons
☐	☒	A full description of the statistical parameters including central tendency (e.g. means) or other basic estimates (e.g. regression coefficient) AND variation (e.g. standard deviation) or associated estimates of uncertainty (e.g. confidence intervals)
☒	☐	For null hypothesis testing, the test statistic (e.g. F, t, r) with confidence intervals, effect sizes, degrees of freedom and P value noted<br>*Give P values as exact values whenever suitable.*
☐	☒	For Bayesian analysis, information on the choice of priors and Markov chain Monte Carlo settings
☐	☒	For hierarchical and complex designs, identification of the appropriate level for tests and full reporting of outcomes
☐	☒	Estimates of effect sizes (e.g. Cohen's d, Pearson's r), indicating how they were calculated

Our web collection on statistics for biologists contains articles on many of the points above.

Software and code

Policy information about availability of computer code

Data collection	Excel, redcap, in house Python based software (icr) for management of medical imaging data
Data analysis	R ver 4.1, Python 3

For manuscripts utilizing custom algorithms or software that are central to the research but not yet described in published literature, software must be made available to editors and reviewers. We strongly encourage code deposition in a community repository (e.g. GitHub). See the Nature Portfolio guidelines for submitting code & software for further information.

Data

Policy information about availability of data

All manuscripts must include a data availability statement. This statement should provide the following information, where applicable:

- Accession codes, unique identifiers, or web links for publicly available datasets
- A description of any restrictions on data availability
- For clinical datasets or third party data, please ensure that the statement adheres to our policy

Data used in this paper is not publicly available due to ethical consideration to maintain participant anonymity

Research involving human participants, their data, or biological material

Policy information about studies with human participants or human data. See also policy information about sex, gender (identity/presentation), and sexual orientation and race, ethnicity and racism.

Reporting on sex and gender	Information on sex is based on self-reported information at the time of recruitment and information on gender identity has not been collected. We have reported the relevant findings, including optimal cut-off for troponin, as well as raw tropnin performance in discriminating clinically actionable CAD status. Sex has been adjusted for in the final model. The sex distribution was fairly equal with 57% male participants.
Reporting on race, ethnicity, or other socially relevant groupings	Majority of participants included in this study are from European ancestory (85%) which has been reported in the manuscript, which is due to the geographical location of patient recruitment. This has been described as one of the limitations of the study in the manuscript. The information regarding race was provided by participants at the time of recruitment, and it has not been as part of statistical analysis. No other social categorisation has been used in this study.
Population characteristics	Mean age of our participants was 61 years, and it was used in the analysis given its confounding effect on all the variables of interest, namely coronary artery disease (specifically CACS), myocardial mass and renal function. Relevant cardiovascular risk factors (diabetes, hypertension, hypercholestraemia and smoking history) have also been included in the study. A special subpopulation of interest in this study is the SMuRFless patients (those who do not have standard cardiovascular risk factors) and as such reporting and accounting for these risk factors was essential.
Recruitment	Patients were recruited at the time of their presentation for clinically indicated CTCA as assessed by their primary physician. Information leaflet was provided at the time of recruitment and informed consent was obtained. A likely source of bias may be language barriers, limiting interaction at the time of recruitment and being unable to provide informed consent.
Ethics oversight	Northern Sydney Local Health District Human Research Ethics Committee - 2019/ETH08376

Note that full information on the approval of the study protocol must also be provided in the manuscript.

Field-specific reporting

Please select the one below that is the best fit for your research. If you are not sure, read the appropriate sections before making your selection.

☒ Life sciences ☐ Behavioural & social sciences ☐ Ecological, evolutionary & environmental sciences

For a reference copy of the document with all sections, see nature.com/documents/nr-reporting-summary-flat.pdf

Life sciences study design

All studies must disclose on these points even when the disclosure is negative.

Sample size	1613 participants from the first 2000 BioHEART participants who met the following inclusion criteria : No prior documented history of cardiomyopathy, myocardial infarction (MI) – defined as previous history of acute coronary syndrome (ACS) – or coronary revascularisation (comprising of percutaneous coronary intervention (PCI) or coronary artery bypass surgery (CABG)); adequate blood sample available; CCTA imaging studies passing quality control. No statistical calculation was performed to predetermine sample size. Current study population size is adequate as it captures the range of underlying CAD seen in general population accurately (as judged by the distribution of CACS and Gensini scores). For smaller subgroups, namely female smurfless participants the sample size may be underpowered however given the low prevalence of CAD in females without standard cardiovascular risk factors, the current sample size remains representative. Dedicated RCT for this subpopulation is warranted in follow up studies.
Data exclusions	The following data exclusions were applied:<br>- Prior MI, PCI or CABG - due to the fact the study is focusing on identifying patients who do not have a known CAD (and hence the need for identifying biomarkers to better triage them) as well as the confounding effect of these on baseline troponin;<br>- Acute presentation or cardiomyopathy: confounding effect on troponin levels<br>- Poor image quality : making accurate assessment of underlying CAD difficult
Replication	No external replication was performed. To assess the reporoducubility of our results, we performed internal validation using bootstrapping and cross-validation
Randomization	No randomisation was performed as this is an observational study. Covariates were included in model development as adjustment terms where appropriate. We also performed subgroup analysis as a means of sensitivity analysis to account for covariates of interest such as sex and symptom status
Blinding	This is an observational study and therefore no blinding was performed.

Reporting for specific materials, systems and methods

We require information from authors about some types of materials, experimental systems and methods used in many studies. Here, indicate whether each material, system or method listed is relevant to your study. If you are not sure if a list item applies to your research, read the appropriate section before selecting a response.

Materials & experimental systems

n/a	Involved in the study
☒	☐ Antibodies
☒	☐ Eukaryotic cell lines
☒	☐ Palaeontology and archaeology
☒	☐ Animals and other organisms
☐	☒ Clinical data
☒	☐ Dual use research of concern
☒	☐ Plants

Methods

n/a	Involved in the study
☒	☐ ChIP-seq
☒	☐ Flow cytometry
☒	☐ MRI-based neuroimaging

Clinical data

Policy information about clinical studies

All manuscripts should comply with the ICMJE guidelines for publication of clinical research and a completed CONSORT checklist must be included with all submissions.

Clinical trial registration	Australian and New Zealand Clinical Trial Registry (ACTRN12618001322224)
Study protocol	Study protocol is available on : https://doi.org/10.1136/bmjopen-2018-028649
Data collection	Outpatient radiology department at Royal Northshore Private Hospital, Sydney, Australia, between 2015 - 2021
Outcomes	Primary outcome was clinically actionable coronary artery disease as identified on CTCA (CACS>100) based on published guidelines

Plants

Seed stocks	*Report on the source of all seed stocks or other plant material used. If applicable, state the seed stock centre and catalogue number. If plant specimens were collected from the field, describe the collection location, date and sampling procedures.*
Novel plant genotypes	*Describe the methods by which all novel plant genotypes were produced. This includes those generated by transgenic approaches, gene editing, chemical/radiation-based mutagenesis and hybridization. For transgenic lines, describe the transformation method, the number of independent lines analyzed and the generation upon which experiments were performed. For gene-edited lines, describe the editor used, the endogenous sequence targeted for editing, the targeting guide RNA sequence (if applicable) and how the editor was applied.*
Authentication	*Describe any authentication procedures for each seed stock used or novel genotype generated. Describe any experiments used to assess the effect of a mutation and, where applicable, how potential secondary effects (e.g. second site T-DNA insertions, mosiacism, off-target gene editing) were examined.*
