# Supplementary material for: Utility of high-sensitivity cardiac troponin I normalized to left ventricular mass in detection of stable coronary artery disease
Source: Nat Cardiovasc Res. 2026 Jul 30;5(8):763–76. doi: 10.1038/s44161-026-00837-z (PMC13461367; doi:10.1038/s44161-026-00837-z)
Supplement: Supplementary file 2 [file 44161_2026_837_MOESM2_ESM.pdf]

nature portfolio

Peer Review File

Utility of High-Sensitivity Cardiac Troponin I Normalised to Left Ventricular Mass in Detection of Stable Coronary Artery Disease

Corresponding Author: Professor Gemma Figtree

This file contains all reviewer reports in order by version, followed by all author rebuttals in order by version.

Version 0:

Reviewer comments:

Reviewer #1

(Remarks to the Author)
I assessed the statistical analyses performed by the authors. Overall, the analysis makes sense to me. I have a few comments below:

1. Line 196: Please provide additional details regarding the “stratified bootstrap resamples.” Was the threshold derived by maximizing Youden’s index re-estimated separately within each bootstrap resample?

2. Line 228: In addressing measurement error within the Bayesian uncertainty modeling, did the authors consider performing sensitivity analyses using different prior assumptions for the measurement error variance? In addition, it would be helpful to clarify whether replicate measurements or external validation data were available to help estimate the variance of measurement error.

3. Figure 3: Please provide more details about the modeling approach, including which specific covariates were included in each model. Also, please clarify whether the confidence intervals shown are 95% CIs.

4. Figure Legends: All error bars should be clearly defined in the corresponding figure legends (e.g., for Figure 3).

5. Figures 4 and Supplementary Figure 5: The marker shapes used in the legends appear inconsistent with those shown in the figures. Please ensure consistency.

6. Figure 6 and Supplementary Figure 1: These figures are not fully self-contained. For example, the type of confidence interval shown in Figure 6 is not described, and the meaning of "***" in Supplementary Figure 1 is not explained. I would also recommend using a shaded region to depict the confidence interval in Figure 6 for improved clarity.

Reviewer #2

(Remarks to the Author)
Retrospective analysis of predicting CACS >100 using HS-TNI screening, with and without adjustment for LVM in 1,613 patients of the BioHEART-CT study. The data on LVM adjustment are original and interesting. On the other hand, the proposed concept is questionable. I would argue a CACS >1 is actionable and the sensitivity of HS-TNI in this setting was 22%. As the authors acknowledged, adjustment for LVM by CCTA makes little sense since with CCTA, one already has detailed CAD assessment. Their model using echo IVS thickness yielded only very modest diagnostic accuracy, however. With further reduction in radiation doses from CACS, we are likely moving towards universal screening for CAD, rendering use of HS-TNI for screening purposes largely superfluous.

Reviewer #3

(Remarks to the Author)

Fathieh and colleagues investigated the value of high-sensitivity troponin I (hs-TnI) to predict coronary CT angiography (CCTA) findings of coronary artery disease in 1,613 individuals undergoing CCTA, suggesting that properly adjusted hs-TnI values may predict CCTA findings and this might prove a potentially cost-effective way to rationalize the use of CCTA, among others.
This is an interesting study based on a large multicenter prospective cohort.
However, the authors need to define more precisely their study population and their independent variable(s) and use more accurate and consistent terminology in the title, the abstract and throughout the text.
Regarding the study population, they actually studied a group of individuals who underwent CCTA either for suspected CAD due to symptoms or for CAD screening in the absence of symptoms. As a result, using terms such as "silent CAD" or "subclinical CAD" are not precise and are rather confusing to the reader.
Regarding the independent variable, it should be clarified in the abstract and throughout the text that it was "clinically actionable CAD" defined as a CACS ≥100 and thus avoid terms such as "CCTA markers of CAD".
The authors adjusted hs-TnI values for age, renal function and LV mass but not for sex obviously because they found hs-TnI to have a similar diagnostic performance in males and females. However, sex-specific cut-offs are widely proposed for the better interpretation of hs-Tn values in terms of diagnosis and prognosis and it might be useful to include sex in the multivariable model.
The authors found that their model performed similarly well both with the CT and the echo-derived LV mass. What was the correlation between the two measurements in patients in whom both were available?
It would be useful to construct a score based on the findings of the multivariate analysis for future validation by other cohorts.
As a minor comment, the authors should better avoid non-established abbreviations and explain all the other abbreviations during their first mention in abstract and throughout the text.

Version 1:

Reviewer comments:

Reviewer #1

(Remarks to the Author)
My comments were well addressed by the authors in the revised manuscript.

Reviewer #2

(Remarks to the Author)
Thank you for your thoughtful responses. While the manuscript has been improved, there are remaining concerns:

The mixing of symptomatic and asymptomatic individuals makes it difficult to assess the value of hsTNI.

Since the manuscript is written with a focus on primary prevention/detection, only asymptomatic individuals should be considered for this analysis. Symptomatic patients will require a CT coronary angiogram anyway.

With such analysis, the NPV may change but, assuming a similar NPV, how do the authors suggest to manage patients with normal hsTNI? It seems not feasible to simply accept that 13% of patients have false negative findings.

The paper would be much stronger if it contained outcome data to validate "actionable" disease in this cohort.

Minor comment: The paper uses the term "CCTA" but only noncontrast data were utilized. So, CCTA is not appropriate in this context.

Reviewer #3

(Remarks to the Author)
The authors have adequately addressed most of my concerns and the manuscript has been improved. I have only a couple of additional comments for consideration:
- In the revised version, the authors characterized their study population as "stable". However, some of the participants were evaluated because of chest pain. But anginal pain is not necessarily stable CAD.
- The AUC for the model to predict CAD was 0,81 when using LV mass-adjusted hs-Tn plus sex (plus additional variables) compared with 0.79 for LV mass-adjusted hs-Tn without sex. Given the known association between sex and LV mass, what would be the predictive value of the model with un-adjusted hs-Tn and sex (plus the additional variables) versus LV mass-adjusted hs-Tn? In other words, if LV mass adjustment controls largely for sex, what is the incremental value of this adjustment – that requires cardiac imaging modality and additional calculations – when simpler just sex-adjusted hs-Tn values might be sufficient for CAD screening?

Version 2:

Reviewer comments:

Reviewer #2

(Remarks to the Author)
The authors acknowledge the limitations of their analysis. Unfortunately, the same limit the impact of their paper. It remains difficult for readers to understand how HS-TNI may be effectively used in clinical practice.

Reviewer #3

(Remarks to the Author)
Thank for considering my additional comments and for addressing them adequately. The manuscript has further been improved. My only remaining concern is that without the direct comparison between the unadjusted hs-cTnI plus sex model and the LV mass-adjusted hs-cTnI model, the clinical relevance of the manuscript remains poor. I feel you could add this analysis although it is not a respecified one, since it came up during the peer review process. In the opposite event, I would stress the mechanistic nature of the study in the title, the abstract and the main text.

Version 3:

Reviewer comments:

Reviewer #3

(Remarks to the Author)
Thank you for addressing my additional concerns. I have no further comments.

Open Access This Peer Review File is licensed under a Creative Commons Attribution 4.0 International License, which permits use, sharing, adaptation, distribution and reproduction in any medium or format, as long as you give appropriate credit to the original author(s) and the source, provide a link to the Creative Commons license, and indicate if changes were made.
In cases where reviewers are anonymous, credit should be given to 'Anonymous Referee' and the source.
The images or other third party material in this Peer Review File are included in the article’s Creative Commons license, unless indicated otherwise in a credit line to the material. If material is not included in the article’s Creative Commons license and your intended use is not permitted by statutory regulation or exceeds the permitted use, you will need to obtain permission directly from the copyright holder.
To view a copy of this license, visit https://creativecommons.org/licenses/by/4.0/

Editorial comments:

In general, we agree with the reviewers that the work would be of interest to the broad audience of Nature Cardiovascular Research. At the same time, the review process identified several opportunities to strengthen the work. We would be happy to look at a revised manuscript that has been revised accordingly (unless something similar has been accepted at Nature Cardiovascular Research or appeared elsewhere in the meantime).

More specifically, we encourage you to improve the presentation of the data and the description of your analyses, as recommended by referee #3. We would also appreciate a detailed response to referee #2, who remained somewhat sceptical about the true utility of this approach when considering that universal screening for CAD may actually be feasible and safe.

We have improved the presentation of the data and description of the analyses as recommended. We have also addressed the suggestions of Referee #2. Whilst we are not against the concept of "universal" CT screening for CAD, we are far from this being a reality in regard to scale and global equity. The suggestion is not dissimilar to suggesting that the "gold standard" colonoscopy would be useful for screening everyone for colon cancer. Furthermore, whilst CCTA is being used in executive health checks as a screening tool, we risk widening the gap between high- and low-SES groups if we pursue such a strategy without a plan for scale and equitable access, particularly in rural and remote areas.

As suggested, we have performed some additional analyses, including robustness/sensitivity analyses (alternative CAD endpoints and measurement-error assumptions), model refinement with sex and prespecified interaction terms, and updated internal validation outputs and figures for clarity and self-containment. These are detailed below

Reviewer #1 (Remarks to the Author):

I assessed the statistical analyses performed by the authors. Overall, the analysis makes sense to me. I have a few comments below:

1. Line 196: Please provide additional details regarding the "stratified bootstrap resamples." Was the threshold derived by maximizing Youden's index re-estimated separately within each bootstrap resample?

Thank you for highlighting the need to make this procedure explicit. We have revised the Statistical Analysis section to provide a step-by-step description of the stratified bootstrap procedure. Briefly, bootstrap resampling was performed within outcome strata (CACS ≥100 vs <100) to preserve the event fraction in each resample. Within each of the 10,000 bootstrap resamples, we identified the hs-cTnI threshold that maximised Youden's J. The distribution of these optimal thresholds across resamples was then summarised using the median (reported as the threshold).

We have updated the text as below – Page 8: Line 195

An optimal hs-cTnI threshold for clinically actionable CAD (CACS ≥ 100) was derived by maximising Youden's index over 10,000 stratified bootstrap resamples with the threshold re-estimated independently in each resample to account for sampling variability and stabilise the estimate the reported threshold is the median across resamples, with percentile-based uncertainty bounds.

Supp. Figure 4 caption is also revised as below:

Supplementary Figure 4. Outcome-stratified bootstrap optimisation of hs-cTnI cutpoint for prediction of CACS ≥100. Outcome-stratified bootstrap resampling (CACS ≥100 vs <100) was performed to preserve class balance. Within each bootstrap resample, the hs-cTnI cutpoint maximising Youden's J (sensitivity + specificity − 1) was re-estimated. Top: density of the resulting optimal cutpoints across resamples (rug marks indicate individual resamples). Bottom: distribution of out-of-bag Youden's J values across resamples.

---

2. Line 228: In addressing measurement error within the Bayesian uncertainty modeling, did the authors consider performing sensitivity analyses using different prior assumptions for the measurement error variance? In addition, it would be helpful to clarify whether replicate measurements or external validation data were available to help estimate the variance of measurement error.

In our Bayesian error-in-variables model, measurement error variance was modelled heteroscedastically to account for the known analytical imprecision of the Abbott ARCHITECT hs-cTnI assay at low concentrations. Specifically, we used a coefficient of variation (CV) of 20% for hs-cTnI levels below 2.5 ng/L (near the assay's limit of quantitation [LoQ] and encompassing many values in our cohort) and 5% for levels at or above 2.5 ng/L. These values were derived from the manufacturer's precision profile and published validation studies for the assay. For instance, Abbott's package insert and independent evaluations (e.g., Beshiri et al., 2017, in Practical Laboratory Medicine) report CVs of approximately 20-33% near the LoD (1.0-1.9 ng/L) and LoQ (3.2-4.7 ng/L), improving to <10% (typically 2-5% within-run and 4-8% between-run) at concentrations

above ~10-20 ng/L, such as around the 99th percentile URLs (14 ng/L for females, 35 ng/L for males, with CVs of 2.7% and 2.0%, respectively) [1-3]. The 2.5 ng/L switch point was selected as a conservative midpoint between the LoD and LoQ, where precision transitions from higher variability (due to signal-to-noise limitations at ultra-low levels) to stable performance, as recommended by IFCC guidelines for hs-cTn assays (≤10% CV at the 99th percentile) [4]. These CVs were converted to log-scale standard deviations using the formula $\sigma\_log = \sqrt{[\log(1 + CV^2)]}$, yielding realistic, concentration-dependent error terms that propagate analytical uncertainty without over- or under-penalising low-level measurements.

Replicate measurements were not performed in the BioHEART-CT cohort, as hs-cTnI was analysed once per participant following standard clinical laboratory protocols to minimise sample volume requirements and align with real-world assay use. Instead, we relied on external validation data from manufacturer precision studies and peer-reviewed literature (e.g., multi-analyser evaluations showing consistent CV profiles across instruments). This approach is common in biomarker modelling when replicates are unavailable, as it leverages established assay characteristics to inform priors.

To evaluate robustness to these assumptions, we conducted sensitivity analyses by varying the CV priors across a range of plausible scenarios. Details are provided here, and a more succinct version in the revised manuscript (see below for revised language included in methods, results, and online supplementary table). :

Base case: CV_low = 20%, CV_high = 5% (as originally modelled).

Conservative (higher imprecision): CV_low = 30%%, CV_high = 7.5% (reflecting potential inter-laboratory variability or sample matrix effects, e.g., as seen in some EDTA plasma studies with CVs up to 33% near LoD) .

Optimistic (lower imprecision): CV_low = 10%, CV_high = 2.5% (aligning with within-run precision at optimal conditions, e.g., 2-4% CVs reported in controlled QC studies) .

Alternative switch point: Shifted to 3.2 ng/L (manufacturer's reported LoQ), with CV_low = 25% and CV_high = 5%.

Across these scenarios, the posterior median AUC remained stable (range: 0.795-0.803; original: 0.799), with negligible changes in calibration (Brier score differences <0.005) and decision-curve net benefit. This confirms that the model's predictive performance is robust to reasonable variations in measurement error assumptions, as the Bayesian framework effectively propagates uncertainty without overfitting to precise low-level values.

We have incorporated these details into the Methods section (Bayesian uncertainty modelling),and in the Results:

Page 10, line 227:

Replicate measurements were not performed in the BioHEART-CT cohort, as hs-cTnI was analysed once per participant following standard clinical laboratory protocols to minimise sample volume requirements and align with real-world assay use. Instead, we relied on external validation data from manufacturer precision studies and peer-reviewed literature

Page 10, line 236:

For sensitivity analysis, we also considered more conservative (reflecting potential inter-laboratory variability or sample matrix effects; CV 30% and 7.5%) and optimistic (aligning with within-run precision at optimal conditions; CV 10% and 2.5%) scenarios.

Page 16, line 376:

We also performed sensitivity analysis using more conservative and optimistic CV values, which showed stable AUC (0.795-0.80) across these values.

---

3. Figure 3: Please provide more details about the modeling approach, including which specific covariates were included in each model. Also, please clarify whether the confidence intervals shown are 95% CIs.

Figure 3 presents **univariable** logistic regression odds ratios; each predictor was assessed independently without covariate adjustment. All confidence intervals represent 95% CIs.

We have updated the caption for clarification:

Univariable logistic regression odds ratios (OR) with 95% confidence intervals (95% CI) for prediction of clinically actionable coronary artery disease. Panel A: CACS ≥100. Panel B: CACS percentile ≥75th percentile (relative to age- and sex- using MESA reference data). Dichotomised hs-cTnI (≥2.3 ng/L vs <2.3 ng/L) is compared with traditional cardiovascular risk factors as binary variables: hypertension, diabetes, smoking history and hypercholesterolaemia. Horizontal bars represent 95% CIs; the vertical dashed line indicates OR = 1.0 (null effect).

---

4. Figure Legends: All error bars should be clearly defined in the corresponding figure legends (e.g., for Figure 3).

We agree and have revised all figure legends to explicitly define error bars.

Figure 3's caption:
Univariable logistic regression odds ratios (OR) with 95% confidence intervals (95% CI) for prediction of clinically actionable coronary artery disease. Panel A: CACS ≥100. Panel B: CACS percentile ≥75th percentile (relative to age- and sex- using MESA reference data). Dichotomised hs-cTnI (≥2.3 ng/L vs <2.3 ng/L) is compared with traditional cardiovascular risk factors. ***Horizontal bars represent 95% CIs****; the vertical dashed line indicates OR = 1.0 (null effect).*

Figure 6's caption:

Decision curve analysis comparing universal versus sequential screening strategies for clinically actionable coronary artery disease (CAD) detection. The net benefit is plotted against the threshold probability (bottom axis) and the corresponding cost-benefit ratio (top axis). The sequential screening strategy (red line)—using an initial adjusted high-sensitivity cardiac troponin I (hs-cTnI) model to triage patients for subsequent CT coronary artery calcium scoring (CACS)—is shown alongside 'treat all' and 'treat none' strategies (grey and

*black lines, respectively). **The error region surrounding the sequential model curve represents the 95% confidence interval derived from 1000 bootstrap resamples.***

Figure 7's caption:

*Internal validation of the Bayesian hs-cTnI model. Left One hundred ROC curves (light blue lines) were generated from random posterior draws of the Bayesian model, with the mean ROC curve overlaid (dark blue line). The dashed diagonal represents chance discrimination (AUC = 0.5). The posterior median AUC was 0.799, demonstrating stable discriminative performance after accounting for measurement error at low hs-cTnI concentrations. Right: Calibration plot by deciles of predicted risk; Observed event rates (y-axis) are plotted against mean predicted probabilities (x-axis) for each decile of predicted risk. Points represent decile means**; vertical error bars represent ±1 standard error of the observed proportion**. The dashed diagonal represents perfect calibration. Close alignment of points with the diagonal indicates good calibration across the spectrum of predicted risk, with no systematic over- or under-prediction.*

---

5. Figures 4 and Supplementary Figure 5: The marker shapes used in the legends appear inconsistent with those shown in the figures. Please ensure consistency.
Thank you for noting this translational error. We have regenerated Figure 4 and Supplementary Figure 5 so that legend keys match the plotted elements, and we have ensured consistency across panels.

---

6. Figure 6 and Supplementary Figure 1: These figures are not fully self-contained. For example, the type of confidence interval shown in Figure 6 is not described, and the meaning of "*" in Supplementary Figure 1 is not explained. I would also recommend using a shaded region to depict the confidence interval in Figure 6 for improved clarity.**

We agree these figures require enhanced legends for self-containment.

We have updated figure 6 to highlight the error CI as shaded region, and updated Figure 6's caption:

*Decision curve analysis comparing universal versus sequential screening strategies for clinically actionable coronary artery disease (CAD) detection. The net benefit is plotted against the threshold probability (bottom axis) and the corresponding cost-benefit ratio (top axis). The sequential screening strategy (red line)—using an initial adjusted high-sensitivity cardiac troponin I (hs-cTnI) model to triage patients for subsequent CT coronary artery calcium scoring (CACS)—is shown alongside 'treat all' and 'treat none' strategies (grey and black lines, respectively). **The error region surrounding the sequential model curve represents the 95% confidence interval derived from 1000 bootstrap resamples.***

It appears that the reviewer's comment in reference to Supp Figure 1 is meant for Supp Table 1; we have now amended this

*Spearman's correlation coefficient of non-transformed hs-cTnI and univariate regression beta coefficient of log-transformed hs-cTnI against related covariates ** denotes p<0.01 and *** denotes p<0.001*

Reviewer #2 (Remarks to the Author):

Retrospective analysis of predicting CACS >100 using HS-TNI screening, with and without adjustment for LVM in 1,613 patients of the BioHEART-CT study. The data on LVM adjustment are original and interesting.

1. **On the other hand, the proposed concept is questionable. I would argue a CACS >1 is actionable and the sensitivity of HS-TNI in this setting was 22%.**

Whilst we agree with Reviewer 2 that any CAD (CACS>0) is evidence of disease presence and susceptibility (as also reflected by the recent changes in the European lipid guidelines), this has not been widely adopted. We selected CACS ≥ 100 as the primary outcome for "clinically actionable" disease based on current major guidelines (e.g., AHA/ACC, Australian Guidelines), and the cut points that trigger specific recommendations for the intensification of pharmacological therapy (such as aspirin and strict LDL targets <1.8 or <1.4 mmol/L). CAC=0 is commonly used to down-classify risk and defer statin therapy in appropriate patients, whereas CAC >100 (or ≥100) is used to up-classify risk and support initiating/intensifying statin therapy (and, in some guidance contexts, consideration of aspirin), as summarised in contemporary global guideline syntheses (Golub et al.; Pavlović et al.). In contrast, CAC 1–99 is more heterogeneous in its downstream management implications and is therefore a less stable definition of “actionable disease” for a triage study.

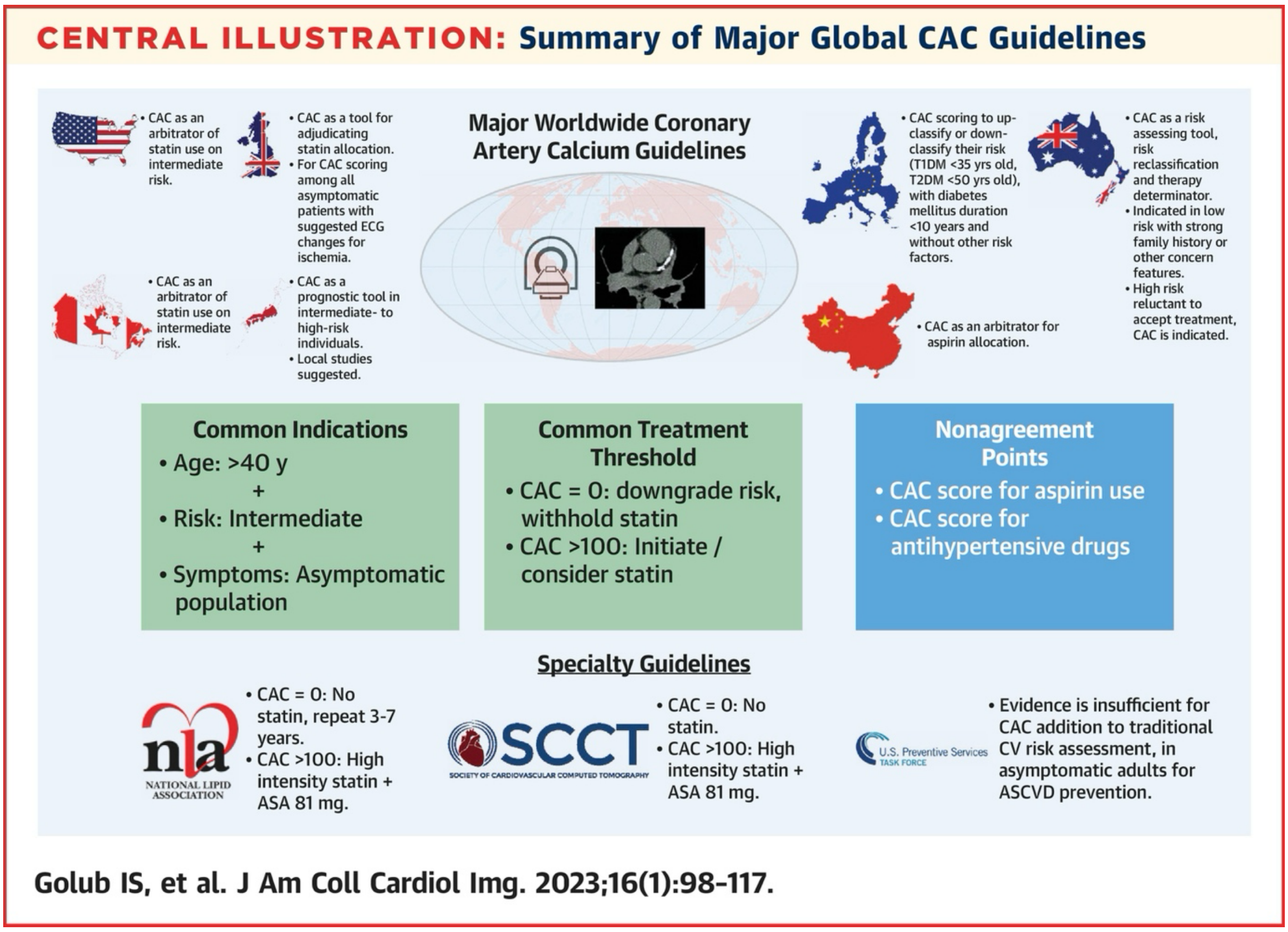

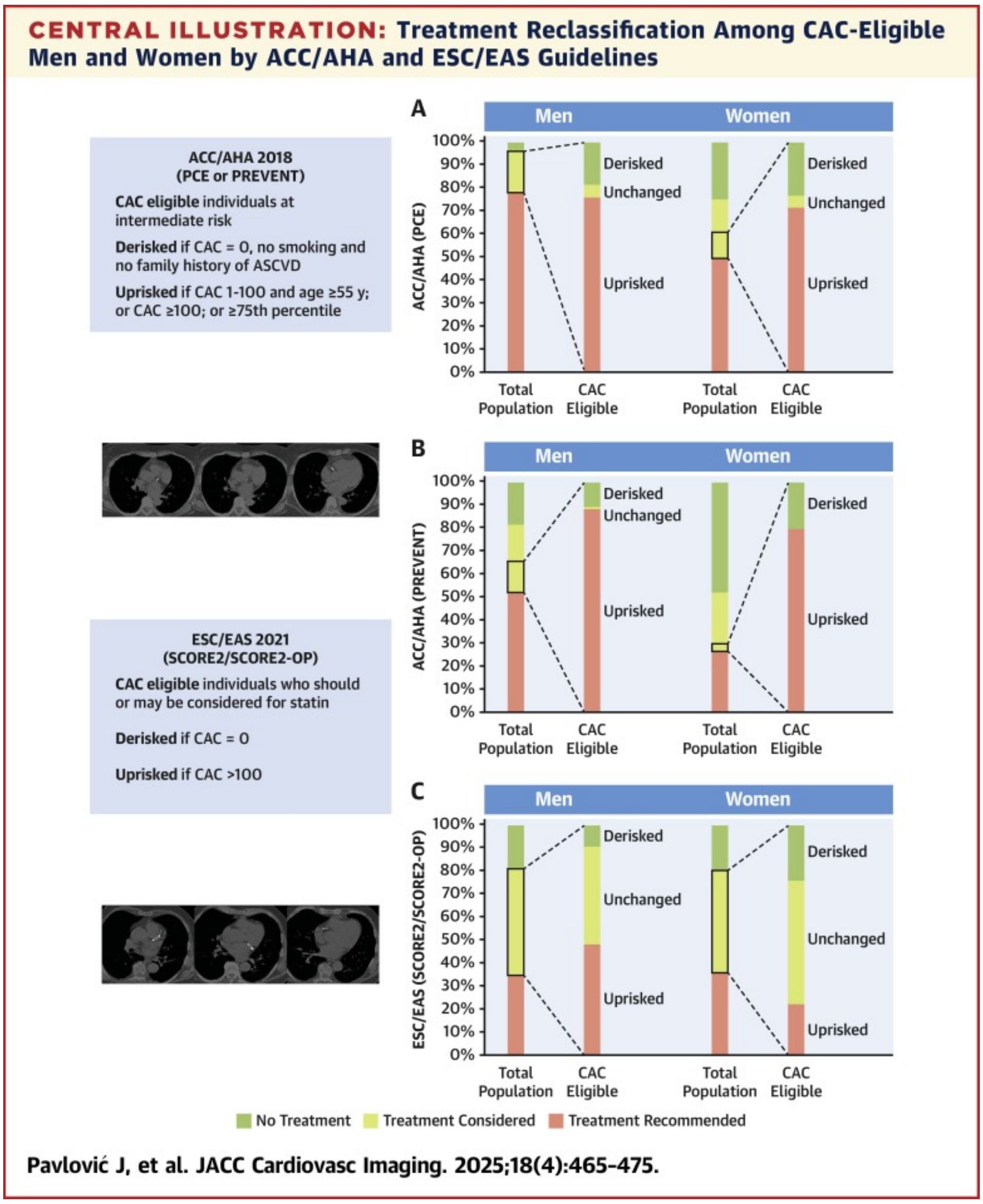

However, as the reviewer notes, the prevalence of CACS > 0 is very high, particularly among older adults. As such, screening for CACS > 0 yields a high positivity rate, potentially reducing the specificity and cost-effectiveness of a triage tool.

In response to the reviewer's comment about the lower sensitivity of hs-TnI for "any" CACS, we would argue that there is a potentially meaningful efficiency signal when hs-cTnI is positioned as a first-stage triage test within a biomarker–imaging pathway. In our two-stage strategy, the number needed to screen to identify one individual with CACS≥1 was 1.03, compared with 1.66 under universal scanning. While this difference may appear modest in absolute terms and may miss some cases of mild atherosclerosis, at the health-system scale, it translates into substantially fewer low-yield scans and a more selective deployment of imaging capacity, with attendant reductions in cost, workflow burden, and downstream testing cascade.

We have added a paragraph to the Discussion section acknowledging the CACS > 0 context and justifying the CACS ≥ 100 choice in accordance with current management guidelines.

Page 7, line 166:

*CAD burden was assessed using the coronary artery calcium score (CACS) as described in the Agatston method 21(hyperattunated areas of at least one mm2 or ≥ three adjacent pixels with >130 Hounsfield units (HU)), **and clinically actionable CAD was defined as CACS ≥ 100 consistent with guideline-endorsed thresholds where intensification of lipid-lowering therapy and, in selected patients, aspirin are recommended 22. Sensitivity analyses were also performed for CACS > 0 and CACS percentile ≥ 75th to capture milder atherosclerosis and age-adjusted burden**.*

Page 14, line 330:

Sensitivity analyses using alternative definitions of CAD (CACS > 0 and CACS percentile ≥ 75th) yielded directionally similar associations between hs-cTnI and plaque burden (Supplementary Table 3).

2- As the authors acknowledged, adjustment for LVM by CCTA makes little sense since with CCTA, one already has detailed CAD assessment.

We agree that using CCTA-derived LVM is not practical in clinical practice (since the diagnosis is already established). CCTA-LVM analysis was initially intended as a mechanistic proof-of-concept to demonstrate why troponin correlates with plaque. We explored the hypothesis that, for the same amount of plaque, more proximal plaque would be associated with higher hs-cTnI (i.e., it is a function of the total downstream myocardial mass). This would be consistent with a mechanism of constant micro-embolic style events. Indeed, our study is the first to show in a stable setting that circulating hs-cTnI reflects low-grade myocardial injury, and the amount of myocardium at risk, downstream of CT-demonstrated CAD, is a key determinant of baseline troponin concentration independent of coronary atherosclerosis.

We realise that the separation of the initial intent (mechanistic insight) and the potential clinical translation/application of LVM (to improve prognostic value of hs-cTnI for an individual) was not as clearly articulated as it could have been. We have now emphasised the two goals. The central message is not "perform CCTA to adjust hs-cTnI", but "interpret hs-cTnI in the context of myocardial mass using whichever modality is available," thereby improving biological interpretability and transportability of thresholds across heterogeneous patient phenotypes.

Page 14, Line 333:

*Given that CT measures of myocardial mass are not pragmatically available (**which is included here as mechanistic insight**), we examined the performance of our latent **score in a clinical application framework** when derived using an echocardiographic measure of LVM (LV septal wall thickness, indexed to BSA). Echocardiographic data were only available for 362 patients, where TTE-derived LVM had a moderate correlation with CT-derived LVM (Pearson's r =0.74, p<0.001). Within this subcohort, AUC showed a promising improvement from baseline of 0.64 to 0.76[0.70-0.811] for the overall cohort and 0.77[0.65-0.89] for the SMuRF-less population (Figure 4).*

3- Their model using echo IVS thickness yielded only very modest diagnostic accuracy, however.

We acknowledge the moderate discriminative performance (AUC 0.76). Several considerations contextualise this finding:

1. **Screening vs. Diagnostic Intent:** For a triage/screening test, the objective is appropriate referral enrichment rather than definitive diagnosis. Our model nearly doubles diagnostic yield per CT examination (0.53 vs 0.29 cases/scan), substantially improving resource efficiency.
2. **Comparison with Established Risk Tools:** The observed AUC compares favourably with existing cardiovascular risk prediction instruments:
   - Framingham Risk Score: AUC ~0.65–0.70
   - SCORE/SCORE2: AUC ~0.70–0.75
   - Adjusted hs-cTnI model: AUC 0.76
3. **Performance in High-Value Populations:** The model demonstrates particular utility in SMuRF-less individuals (AUC 0.77, sensitivity 90%)—a population inadequately served by conventional risk algorithms (e.g. Framingham Risk Score AUC; SCORE/SCORE2 AUC-) and representing a substantial proportion of first-presentation myocardial infarction patients.

Page 14,line 333:

Given that CT measures of myocardial mass are not pragmatically available (which is included here as mechanistic insight), we examined the performance of our latent score in a clinical application framework when derived using an echocardiographic measure of LVM (LV septal wall thickness, indexed to BSA). Echocardiographic data were only available for 362 patients, where TTE-derived LVM had a moderate correlation with CT-derived LVM (Pearson's r =0.74, p<0.001). Within this subcohort, AUC showed a promising improvement from baseline of 0.64 to 0.76[0.70-0.811] for the overall cohort and 0.77[0.65-0.89] for the SMuRF-less population (Figure 4).

With further reduction in radiation doses from CACS, we are likely moving towards universal screening for CAD, rendering use of HS-TNI for screening purposes largely superfluous.

This perspective on evolving CAD screening paradigms merits thoughtful consideration. Indeed, senior author Figtree has published a similarly thought-provoking review and perspective with other clinical leaders in the field ("Examining the potential of coronary artery calcium scoring for individuals at low cardiovascular risk- Heart Lung Circ. 2021; 30(12): 1819-1828). In response to the Referee, we now discuss this possibility, as well as the practical benefits of a triage approach to achieve scale and equity:

1. **Global Healthcare Context:** Despite reduced CACS radiation doses (~0.5–1 mSv), and some evidence for value of CAC screening even in apparently "low-risk" individuals" (ref above- and others?), universal CT screening remains impractical in most healthcare systems due to infrastructure costs, scanner availability, radiographer workforce constraints, and healthcare system capacity limitations. A blood-based screening approach, triaging to imaging, offers opportunities for scalability, particularly in resource-limited, or geographically remote settings, where cardiovascular disease burden and clinical outcomes are the greatest challenge.

2. **Sequential Strategy Value:** Even where CACS is available, a sequential approach (hs-cTnI triage → CACS for screen-positives) reduces CT examinations by ~59% while maintaining 87% NPV for CAD absence, representing substantial potential resource savings.

3. **Integration with Routine Care:** hs-cTnI can be incorporated into routine blood-based health assessments without additional visits, enabling passive population surveillance not achievable with imaging-based approaches.

4. **Future Applications:** hs-cTnI may serve as one component of emerging multi-marker panels that could further improve discrimination beyond what either marker achieves alone.

Please refer to Figures 5 and 6, as well as Table 4, for a direct comparison between the universal imaging strategy and sequential screening. The provided data clearly demonstrate the benefits of adopting a more targeted approach.

Table 4 - Comparison of CAD Screening Strategies

Metric	Universal Screening	Sequential Screening
Sensitivity	100%	76 %
Specificity	-	75 %
Scans Performed	1,575	619
Scans Reduction (%)	0%	61%
Missed Diagnosis (%)	0%	114
NNS	3.4	Stage 1= 4.47<br>Stage 2=1.8
Diagnostic Yield (cases per scan)	0.29	0.57
LR+	-	3.04
LR-	-	0.32
DOR	-	9.5
Bayesian PPV	-	0.56
Bayesian NPV	-	0.88

We have added the following paragraph on page 17, line 396:

While we acknowledge the trajectory towards lower radiation-dose CACS, this does not, in itself, imply that universal CT-based screening will become feasible or appropriate at scale. Even at contemporary doses (~0.5–1 mSv), population-wide CACS would require substantial expansion in scanner capacity, radiographer workforce, and downstream clinical pathways,

with inevitable opportunity costs and inequities across resource-limited or geographically remote settings. As highlighted in prior perspectives from our group and others, CAC may ultimately play an expanded role even in apparently low-risk individuals 29; however, the central implementation challenge is not radiation alone, but scalability, access, and health-system throughput. In that context, a blood-based triage step retains practical and clinical value: in our data, an hs-cTnI-based sequential strategy reduced CT examinations by ~59–60% while maintaining an NPV of ~87% for excluding clinically actionable CAD, thereby enriching imaging yield and potentially enabling more equitable deployment of CT resources. We therefore view hs-cTnI not as a competitor to CACS, but as a complementary upstream filter that can help operationalise targeted imaging strategies—particularly in SMuRF-less and otherwise "low-risk" groups where current primary prevention algorithms underperform.

The following reference was added:

Playford D, Hamilton-Craig C, Dwivedi G, Figtree G. Examining the Potential for Coronary Artery Calcium (CAC) Scoring for Individuals at Low Cardiovascular Risk. Heart Lung Circ. 2021 Dec;30(12):1819-1828. doi: 10.1016/j.hlc.2021.04.026. Epub 2021 Jul 29. PMID: 34332891.

Reviewer #3 (Remarks to the Author):

Fathieh and colleagues investigated the value of high-sensitivity troponin I (hs-TnI) to predict coronary CT angiography (CCTA) findings of coronary artery disease in 1,613 individuals undergoing CCTA, suggesting that properly adjusted hs-TnI values may predict CCTA findings and this might prove a potentially cost-effective way to rationalize the use of CCTA, among others.
This is an interesting study based on a large multicenter prospective cohort. However, the authors need to define more precisely their study population and their independent variable(s) and use more accurate and consistent terminology in the title, the abstract and throughout the text

We appreciate the reviewer's recognition of our study's strengths and their suggestions for precision in terminology and analysis. We have revised the manuscript accordingly.

.

1- Regarding the study population, they actually studied a group of individuals who underwent CCTA either for suspected CAD due to symptoms or for CAD screening in the absence of symptoms. As a result, using terms such as "silent CAD" or "subclinical CAD" are not precise and are rather confusing to the reader.

We have revised the Title, Abstract and main text to define the cohort as stable outpatients referred for CCTA either for symptoms or elective screening, without acute coronary syndrome. We have removed or tightened ambiguous terms ("silent CAD", "CCTA markers") and now use consistent terminology throughout. We now refer to "stable chest pain" and "asymptomatic screening" subgroups where appropriate. We have replaced broad references to "silent CAD" with "stable CAD" where specifically discussing the asymptomatic sub-cohort.

Revised title:

Utility of High-Sensitivity Cardiac Troponin I Normalised to Left Ventricular Mass in Detection of Stable Coronary Artery Disease

Instances of **silent CAD** or **subclinical CAD** that were replaced with **stable CAD**:

Line 43 : *as a non-invasive biomarker to screen for stable, clinically actionable CAD*

Line 68 : *cost-effective biomarker for stable CAD (symptomatic or asymptomatic CAD patients without an acute event).*

Line 88 : *Hs-cTnI is a promising screening tool for detecting stable CAD.*

Line 92: *Adjusting for LV myocardial mass, age, and renal function further boosts the biomarker's predictive power for significant stable CAD*

Line 95: *... where troponin is no longer read as a crude, absolute value but as an individualised signal of stable CAD risk ...*

Line 117: *The burden of stable CAD (patients with detectable CAD on imaging, with and without symptoms who have not had an acute event)*

Line 121: *... stable CAD was common (42.1%) ...*

Line 136: *... , particularly in vulnerable populations, including those with stable CAD and SMuRFless, ...*

Line 257: *However, this likely includes a substantial number with stable CAD.*

Line 460: *... , and particularly the need for diagnostic pathways to help pick up CAD in its stable phase. Providing equitable access to clinical pathways for detecting and treating stable CAD before a heart attack has the potential to reduce cardiovascular morbidity and mortality dramatically.*

Regarding the independent variable, it should be clarified in the abstract and throughout the text that it was "clinically actionable CAD" defined as a CACS ≥100 and thus avoid terms such as "CCTA markers of CAD".

We have adopted this suggestion throughout the abstract and results section, to state explicitly that the primary outcome is clinically actionable CAD defined a priori as CACS ≥100, and we have reframed other imaging metrics as secondary/sensitivity endpoints.

Line 43, we have explicitly added the definition for clinically actionable CAD:

.., clinically actionable CAD (CACS ≥100).

Line 45:

We investigated the associations between hs-cTnI and clinically actionable CAD as primary outcome , and CCTA markers of CAD as secondary endpoints, in a stable cohort of 1,613 adults referred for coronary CT angiography (CCTA).

Line 55:

Hs-cTnI was positively associated with our primary and secondary endpoints of interest.

Line 69:

Embedding this score into a two-stage screening pathway substantially reduces the number of scans required to detect clinically actionable CAD ...

Line 88:

A threshold of >2.3 ng/L yields a clinically acceptable predictive value for detection of clinically actionable CAD.

Line 136:

In this study, we aim to investigate the utility of hs-cTnI in detecting CCTA-determined clinically actionable CAD, ...

The authors adjusted hs-TnI values for age, renal function and LV mass but not for sex obviously because they found hs-TnI to have a similar diagnostic performance in males

and females. However, sex-specific cut-offs are widely proposed for the better interpretation of hs-Tn values in terms of diagnosis and prognosis and it might be useful to include sex in the multivariable model.

Our initial decision to exclude sex as a separate covariate was predicated on the hypothesis that sex differences in hs-cTnI are predominantly mediated through myocardial mass differences (males have ~30% larger LVM on average). By adjusting for LVM directly, we reasoned that the primary biological driver of sex differences would be accounted for. Supporting this hypothesis:

- Subgroup analysis demonstrated equivalent AUC in males (0.62) and females (0.62)
- Optimal thresholds were nearly identical (males: 2.38 ng/L; females: 2.31 ng/L)

However, the addition of sex to the LVM-indexed model did improve the AUC from 0.79 to 0.81 and as such, we have updated the Methods and Results clarifying this finding:

The following line was removed:

Sex was not separately adjusted, as its effect on hs-cTnI is likely mediated predominantly through differences in myocardial mass.

The following lines were adjusted:

Page 9, line 203:

Model 3: Model 2 + renal function (eGFR), sex and prespecified interaction terms to account for the effect of age on all variables (both on the dependent variable, risk of CAD, and independent variables) and the interaction between sex and LVM

Page 14, line 320:

Model 3: Accounting for Renal Clearance—Adding serum creatinine as a measure of renal clearance, while accounting for the impact of age and sex further enhanced the model (ΔAUC = 0.16, p <0.001), indicating an independent predictive value of renal function, as well as the known effect of age and sex. We will refer to this model as the "indexed-troponin model".

Page 15, line 345:

It demonstrated comparable performance in both groups (ROC-AUC in symptomatic patients was 81.5% [78.3-84.6] versus 81.8% [78.5-81.8] in asymptomatic patients).

In the Utility in Clinical Practice section the following values were corrected based on the new model:

- Diagnostic yield : 0.57 (from 0.53)
- NNS: 1.8 (from 1.9)
- PPV: 56% (from 53%)
- NPV: 87% (from 88%)
- SMuRFless NNS:3 (from 3.2)

Figure 4's AUC has been updated to 0.81 for CT derived LVM (left panel)

Figure 5 has also been updated with the new positive and negative cases per CACS subcategory

Tables 3 and 4 also updated with the new results for model 3

The authors found that their model performed similarly well both with the CT and the echo-derived LV mass. What was the correlation between the two measurements in patients in whom both were available?

In the subset of patients with both measurements (n=362), the Pearson's correlation between CCTA-derived LVM and Echo-derived LVM was moderate to strong (0.74, p<0.001). We have added this correlation coefficient to the Results section to validate the bridge between the two modalities.

Page 14, line 336:

Echocardiographic data were only available for 362 patients where TTE derived LVM had a moderate correlation with CT derived LVM (Pearson's p =0.74, p<0.001).

It would be useful to construct a score based on the findings of the multivariate analysis for future validation by other cohorts.
We thank the reviewer for this constructive suggestion, which aligns with our long-term translational objectives of integrating hs-cTnI into clinical decision making for stable patients to improve early detection and treatment of CAD. However, we felt it was premature to include a formal risk score in the present manuscript. We have therefore prioritised reporting the full set of continuous model coefficients (Supplementary Table 4), enabling independent investigators to implement and externally validate the model in their own cohorts.

Page 14, line 324:

Regression coefficients for Model 3, both with dichotomised and also continuous hs-cTnI are included in Supplementary Table 4.

As a minor comment, the authors should better avoid non-established abbreviations and explain all the other abbreviations during their first mention in abstract and throughout the text.

We have carefully audited the manuscript to address this valid concern.

- Non-established terms: We have removed niche abbreviations such as 'NCPS' (replacing it with 'non-calcified plaque score') and added a plain English description of technical statistical abbreviations like 'PSIS-LOO' to improve readability for the clinical audience.
    - Line 247: *PSIS-LOO is a cross-validation approximation for assessing out-of-sample predictive performance*
    - Line 273, 283, 405: *non-calcified plaque score*
    - Line 372 : *only two observations showing mildly elevated Pareto-k values - which flags influential observations-*

- Clarification: We have ensured that all standard abbreviations (e.g., CACS, TTE, BSA, eGFR) are explicitly defined upon their first appearance in both the Abstract and the main body of the text.

    - Line 43 : *coronary artery calcium score - CACS*
    - Line 182. : *Transthoracic echocardiography (TTE)*
    - Line 203: *estimated Glomerular Filtration Rate - eGFR*
    - Line 263 : *body surface area (BSA)*
    - Line 640 : *Multi-Ethnic Study of Atherosclerosis (MESA).*
    - Figure 4 caption and line 340 : *Receiver Operating Characteristic Area Under the Curve*

Reviewer #1 (Remarks to the Author):

My comments were well addressed by the authors in the revised manuscript.

We thank the reviewer for their input and positive feedback.

Reviewer #2 (Remarks to the Author):

Thank you for your thoughtful responses. While the manuscript has been improved, there are remaining concerns:

The mixing of symptomatic and asymptomatic individuals makes it difficult to assess the value of hsTNI.

Since the manuscript is written with a focus on primary prevention/detection, only asymptomatic individuals should be considered for this analysis. Symptomatic patients will require a CT coronary angiogram anyway.

Our cohort is a pragmatic real-world population of stable patients (considered clinically not to be acute coronary syndrome patients) in which referral decisions are often made under symptom uncertainty, particularly in patients with atypical symptoms, anginal equivalents, or with no risk factors, for screening purposes, particularly in the setting of discordant risk profiles. The previously published BioHEART protocol describes the discovery and validation of biomarkers in this group.

We agree that, if the intended application is framed strictly as primary prevention screening, the asymptomatic subgroup (45% of the total cohort) is the most directly relevant population. We have therefore revised the manuscript to make clear that the primary-prevention implications of our findings are supported principally by the asymptomatic subgroup analysis.

Regarding the symptomatic stable population, we are very sensitive to the limitations of clinical assessment of potential ischaemia in practice, with shortness of breath and chest pain not mapping cleanly onto atherosclerotic burden. Taking this into consideration, a biomarker such as hs-cTnI may complement symptom-based triage rather than replace it.

Importantly, to directly address the Reviewer's concern, our included symptom-stratified analyses found that the indexed hs-cTnI model showed comparable discrimination in symptomatic and asymptomatic subgroups (ROC-AUC 81.5% [78.3–84.6] versus 81.8% [78.5–81.8], respectively).

1- 2024 ESC Guidelines for the management of chronic coronary syndromes: Developed by the task force for the management of chronic coronary syndromes of the European Society of Cardiology (ESC) Endorsed by the European Association for Cardio-Thoracic Surgery (EACTS), European Heart Journal, Volume 45, Issue 36, 21 September 2024, Pages 3415–3537, https://doi.org/10.1093/eurheartj/ehae177
2- Pretest probability for patients with suspected obstructive coronary artery disease: re-evaluating Diamond–Forrester for the contemporary era and clinical implications: insights from the PROMISE trial, European Heart Journal - Cardiovascular Imaging, Volume 20, Issue 5, May 2019, Pages 574–581

3- Ford TJ, Berry C. Angina: contemporary diagnosis and management. Heart. 2020 Mar;106(5):387-398. doi: 10.1136/heartjnl-2018-314661. Epub 2020 Feb 12. PMID: 32054665; PMCID: PMC7035719.

We have now revised the manuscript to emphasise that the asymptomatic subgroup provides the clearest support for screening, while the mixed cohort illustrates potential utility in real-world settings where symptom classification is often uncertain.

Page 16, line 342:

In this pragmatic cohort study, 55% of patients had been referred for stable symptoms, and 45% were asymptomatic; "screening" studies were patient-driven and not reimbursed by the government. Of interest, a higher plaque burden was observed in the asymptomatic sub-cohort, as shown in Supplementary Table 2. We then performed a sensitivity analysis to test the predictive performance of the adjusted cTnI in the asymptomatic sub-cohort. It demonstrated comparable performance in both groups (ROC-AUC in symptomatic patients was 81.5% [78.3-84.6] versus 81.8% [78.5-81.8] in asymptomatic patients).

Page 19, line 425

Although symptom status is central to current diagnostic pathways, it is an imperfect surrogate for underlying coronary atherosclerotic burden. Contemporary evidence has shown that traditional age-sex-symptom pre-test probability models substantially overestimate obstructive CAD in modern populations undergoing CT evaluation, and recent guidelines now favour risk factor-weighted clinical likelihood rather than symptom category alone 30. In parallel, a substantial proportion of patients presenting with angina symptoms do not have obstructive epicardial CAD, reflecting alternative mechanisms such as microvascular or vasospastic disease, or non-cardiac origins. Within this context, an objective blood-based biomarker may provide complementary value by refining triage when symptoms are absent, atypical, or of uncertain significance.

Page 22, line 495

Second, the study cohort was not a pure primary-prevention population, but a pragmatic real-world mixture of patients referred with stable symptoms suspicious for CAD or asymptomatic individuals referred for screening. While this limits the extent to which the full cohort alone can define the role of hs-cTnI specifically as a screening biomarker, it also reflects the reality that symptom-based referral pathways are frequently heterogeneous and clinically uncertain. Strong performance demonstrated by the sensitivity analysis in the asymptomatic subgroup provides the most direct support for a potential screening application of hs-cTnI, whereas the combined cohort informs potential pragmatic utility in contemporary referral practice.

With such analysis, the NPV may change but, assuming a similar NPV, how do the authors suggest to manage patients with normal hsTNI? It seems not feasible to simply accept that 13% of patients have false negative findings.

We thank the Reviewer for this important point and agree that a normal hs-cTnI result cannot be interpreted as definitively excluding clinically actionable CAD. We do not propose hs-cTnI as a stand-alone rule-out test. Rather, we propose it as a first-line triage tool within a multistep prevention pathway intended to improve the yield of downstream CT imaging while reducing unnecessary scans.

Accordingly, patients with normal hs-cTnI would not be considered "cleared" of disease. Instead, a normal result would support risk down-classification only, with continued guideline-based preventive care, periodic reassessment, and escalation to coronary imaging when symptoms, absolute risk profile, family history, or complementary biomarkers indicate persistent concern.

We agree that the false-negative proportion observed in our retrospective analysis is not negligible. This trade-off reflects the balance inherent in any staged screening strategy between sensitivity and resource utilisation, and it is threshold-dependent. A future implementation pathway would likely use thresholds tailored to the intended population and objective, including more sensitivity-favouring thresholds where minimising missed disease is paramount. In addition, hs-cTnI could be combined with complementary markers to further improve first-stage sensitivity. For example, in prior work from our group (1), Lp(a) provided additional stratification of subclinical CAD burden, supporting a multi-marker enrichment strategy rather than relying on a single blood test.

1- S. Fathieh, et al, Evaluating the role of lipoprotein(a) in enhancing risk stratification for the presence and extent of subclinical coronary artery disease burden: a BioHEART-CT study, EJPC, 2025; https://doi.org/10.1093/eurjpc/zwaf323).

We have revised the manuscript to make clear that hs-cTnI should be viewed as an upstream enrichment tool within a personalised screening pathway, and that a normal hs-cTnI should not be interpreted as definitive exclusion of CAD.

Page 18, line 411

It is worth highlighting that a normal hs-cTnI in this framework should not be interpreted as excluding clinically actionable CAD. Rather, hs-cTnI is better conceptualised as an upstream enrichment tool to improve allocation of downstream CT resources. Individuals with a low hs-cTnI-based score would still warrant ongoing guideline-based preventive care and could undergo repeat biomarker assessment or coronary imaging if their overall risk profile, family history, symptom trajectory, or other clinical features remained concerning. Thus, the proposed role of hs-cTnI is one of risk down-classification, not absolute rule-out. Furthermore, the balance between missed cases and scan reduction is inherently threshold-dependent, and future prospective implementation studies should determine the threshold that best matches the intended clinical objective, with more sensitivity-favouring thresholds likely appropriate when minimising missed disease is prioritised.

Page 23, line 520

Finally, the false-negative proportion observed in the current retrospective modelling indicates that hs-cTnI should not be used in isolation to exclude CAD, but rather as one component of a broader, longitudinal risk-stratification strategy and a decision aid regarding the use of CT imaging.

The paper would be much stronger if it contained outcome data to validate "actionable" disease in this cohort.

We agree with the Reviewer that prospective outcome data would materially strengthen the manuscript and provide the most rigorous validation of the term "actionable" disease. At present, medium- to long-term event follow-up within BioHEART-CT is still maturing, and these analyses are planned as a future extension of this work.

However, we would respectfully clarify that, in the current study, "actionable" was not intended to imply that we have directly validated this endpoint against clinical events within this cohort. Rather, we used this term to denote imaging-defined CAD phenotypes that are currently referred to in practice guidelines and thresholds used to guide changes in clinical management, including intensification of preventive therapies and consideration of further investigation

To address the Reviewer's concern, we have revised the manuscript to make this distinction explicit and to more clearly acknowledge, in the Limitations and Discussion, that outcome-based validation remains an important next step.

Page 22, line 505

Thirdly, our study endpoint of "clinically actionable" CAD is based on CT-detection of atherosclerosis above a threshold for treatment according to clinical guidelines. The BioHEART study is not yet powered for validation against clinical outcomes such as MI. Fourthly, our study is a retrospective study, and prospective testing is warranted. We would advocate for a rapid translational implementation study akin to the ESCALATE study to evaluate whether the LVM-adjusted hs-cTnI could improve the detection of subclinical CAD in primary care 42. If positive, testing the clinical utility of hs-cTnI-based enrichment for CTCA to impact treatment and reduce MI would be warranted.

Minor comment: The paper uses the term "CCTA" but only noncontrast data were utilized. So, CCTA is not appropriate in this context.

We agree that the wording in parts of the manuscript may have insufficiently distinguished between the primary modelling endpoint, which was based on CACS and the broader imaging dataset, which also included contrast-enhanced CTCA-derived measures. The manuscript includes several genuine CCTA-derived analyses, including CT-derived left ventricular mass (which relied on the contrast enhancement, the higher resolution and thinner slices available in CCTA), segmental plaque distribution (allowing us to model the downstream impact of stenosis on background hs-cTnI

leakage), and quantitative plaque burden measures such as non-calcified plaque score and Gensini stenosis score. However, for the specific sequential screening model intended as a pragmatic first-stage triage strategy, the primary endpoint was intentionally defined using CACS ≥100, derived from the non-contrast component of the CT examination.

Accordingly, we have amended terminology where needed so that references to "CCTA-determined" disease are reserved for the relevant secondary analyses, while the main screening endpoint is described more precisely as CT calcium score-defined or non-contrast CT-defined clinically actionable CAD.

Page 6, line 136

In this study, we aimed to investigate the utility of hs-cTnI in detecting CT-defined clinically actionable CAD, primarily defined by coronary artery calcium burden, while also examining its relationship with secondary CCTA-derived measures of plaque burden and myocardial mass.

Reviewer #3 (Remarks to the Author):

The authors have adequately addressed most of my concerns and the manuscript has been improved. I have only a couple of additional comments for consideration:

- In the revised version, the authors characterized their study population as "stable". However, some of the participants were evaluated because of chest pain. But anginal pain is not necessarily stable CAD.

We thank the Reviewer for this important comment and agree that the term "stable" may have been used too broadly in the revised manuscript. We now describe the study population as a ***suspected chronic coronary syndrome*** or ***outpatient non-acute cohort***, comprising both asymptomatic screening referrals and individuals referred for evaluation of suspected CAD/chest pain in the absence of acute coronary syndrome.

Instances update in:

- Page2, line 46
- Page 3, line 69
- Clinical perspective (page 5, lines 90, 95, 97)
- Page 6 lines 119, 123, 130
- Page 12, line 270
- Page 19, line 435
- Page 20, line 447, 462
- Page 21, lines 470, 472, 482, 488
- Page 23, lines 524,525

- The AUC for the model to predict CAD was 0,81 when using LV mass-adjusted hs-Tn plus sex (plus additional variables) compared with 0.79 for LV mass-adjusted hs-Tn without sex. Given the known association between sex and LV mass, what would be the predictive value of the model with un-adjusted hs-Tn and sex (plus the additional variables) versus LV mass-adjusted hs-Tn? In other words, if LV mass adjustment controls largely for sex, what is the incremental value of this adjustment – that requires cardiac imaging modality and additional calculations – when simpler just sex-adjusted hs-Tn values might be sufficient for CAD screening?

We agree that an important question is whether the apparent benefit of LV mass adjustment simply reflects sex-related differences in myocardial size, and whether a simpler model using unadjusted hs-cTnI with sex adjustment might provide comparable performance. However, in our current analysis, the incremental value of myocardial mass was evident before sex was added to the model. Specifically, incorporating the hs-cTnI×LVM interaction increased discrimination from an AUC of 0.624 for hs-cTnI alone to 0.644, with a statistically significant interaction term ($p=0.012$), whereas sex was introduced only in the subsequent model together with renal function and age interactions. This suggests that LV mass is not acting solely as a proxy for sex, but is

capturing biologically relevant variation in the relationship between circulating troponin and the burden of coronary disease.

This interpretation is also supported by the subgroup analyses, in which the optimal unadjusted hs-cTnI thresholds were very similar in men and women (2.38 ng/L and 2.31 ng/L, respectively), with comparable discrimination in both sexes. These findings suggest that simple sex stratification alone may not fully account for the myocardial-mass-related component of the hs-cTnI signal.

That said, we agree that a direct comparison between (i) unadjusted hs-cTnI plus sex and the same clinical covariates, and (ii) LV mass-adjusted hs-cTnI models, would be informative. This was not a prespecified analysis in the current manuscript, which was designed to test a mechanistic hypothesis regarding downstream myocardial mass. We have therefore added text to the Discussion acknowledging this as an important additional comparison for future work and clarifying that the principal contribution of LV mass adjustment in the present study is to provide mechanistic insight, while echocardiographic surrogates may offer a more pragmatic route to implementation.

Page 20, line 445
An important methodological question is whether the effect of LV mass adjustment simply reflects sex-related differences in myocardial size. In our analysis, however, the incremental improvement associated with the hs-cTnI×LVM interaction was evident before sex was introduced into the subsequent model, and the optimal unadjusted hs-cTnI thresholds were very similar in men and women. Together, these findings suggest that myocardial mass is not acting solely as a proxy for sex but may capture biologically relevant variation in the amount of downstream myocardium contributing to circulating troponin.

Reviewer #2 (Remarks to the Author):

The authors acknowledge the limitations of their analysis. Unfortunately, the same limit the impact of their paper. It remains difficult for readers to understand how HS-TNI may be effectively used in clinical practice.

Whilst acknowledging limitations (as appropriate), we believe that these do not counter the novel findings that have substantial and imminent translational implications, and the potential for substantial clinical impact. To make this clearer, we have revised the discussion and highlighted the translational steps we believe are immediately feasible. This includes a prospective clinical study we are currently finalising with international experts, building on our recently completed ESCALATE study that used a polygenic risk score in primary care.
The demonstration that hs-trop (taken immediately prior to the clinical CT scan in the "real world") can enrich apparently low-risk populations for the presence of clinically actionable CAD has direct translational significance, addressing a specific unmet need raised by both the Lancet Commission for Rethinking CAD and the international CAD Frontiers consortium.

Page 21, line 489
Our results show that hs-cTnI can enrich apparently low-risk non-acute populations for the presence of clinically actionable CAD, known to be associated with a high rate of myocardial infarction 42, and drive intensive medical treatment as per new guidelines 43,44. This may be particularly valuable in those who develop CAD despite low traditional risk factor burden ("SMuRFless") 4 who do not meet criteria for preventative therapy or screening CT scan. Whilst the use of hs-cTnI is not diagnostic in this stable population, using levels above X to guide screening CT decisions has the potential to improve the yield of this powerful and accurate imaging tool, with implications for scale and equity. Data presented in this study support the value of a prospective study, using hs-cTnI ≥ 2.3 to triage individuals for CT. Similar to the study design we have implemented for CAD polygenic risk scoring in the ESCALATE Study 45 (recruitment closed, analysis in progress), the prognostic power of hs-cTnI could be prospectively tested in primary care to see if it can reduce the number of CT scans needed to detect patients with clinically actionable CAD, and lead to appropriate intensive medical treatment in those shown to have the disease. Longer term, larger studies could consider the impact of implementing this hs-cTnI-CT decision pathway on plaque progression and ultimately heart attack prevention.

Page 18, line 399
Although the demonstrated value of LVM adjustment is consistent with the hypothesis that myocardial volume contributes to low-level hs-cTnI release, a simpler hs-cTnI-by-sex model performed similarly to the hs-cTnI-by-LV mass model. This likely relates to the very strong relationship of sex and LVM (noting that when sex was included in LVM in the model, it was not a significant contributor). This has practical implications, suggesting that sex and BMI/BSA could be used to replace LVM in clinical settings where LVM is unavailable.

Reviewer #3 (Remarks to the Author):

Thank for considering my additional comments and for addressing them adequately. The manuscript has further been improved. My only remaining concern is that without the direct comparison between the unadjusted hs-cTnI plus sex model and the LV mass-adjusted hs-cTnI model, the clinical relevance of the manuscript remains poor. I feel you could add this analysis although it is not a prespecified one, since it came up during the peer review process.

We thank the Reviewer and acknowledge that some classification metrics were similar between models. We have therefore revised the manuscript to present LV mass adjustment as providing incremental mechanistic and modelling value beyond sex alone, rather than implying that sex adjustment is uninformative. We have added this sensitivity analysis to the manuscript:

Page 2, line 60

hs-cTnI (ρs = 0.32, p <0.001) was proportional to LVM in both healthy and CAD patients. Indexing hs-cTnI for age, LVM, and renal function improved its predictive ability for CAD, with the AUC reaching 0.8 (posterior median AUC of 5-fold CV = 79.9% (95% CI 79.6-80.0)). Sex, strongly associated with LVM, also improved the model's prognostic performance for CAD.

Page 14, line 320

It is worth noting that in a post hoc sensitivity analysis, given the strong correlation between sex and LVM, a model incorporating the interaction between hs-cTnI and sex demonstrated similar discrimination to the corresponding hs-cTnI-by-LV mass model (ROC-AUC 0.65 [95% CI 0.630–0.686] versus 0.65 [0.615–0.674]; DeLong p=0.13) while the LVM interaction model demonstrated superior overall model fit, with lower AIC (1828.2 vs 1848.1) and BIC (1849.7 vs 1869.6 In a subgroup analysis of males, LVM remained as a significant independent variable. Both sex and BMI become insignificant in a model that also includes LVM, indicating that in the absence of LVM measurements, surrogate markers such as sex may be useful, but myocardial mass captures information beyond crude sex stratification alone.

In the opposite event, I would stress the mechanistic nature of the study in the title, the abstract and the main text.

We have adjusted the abstract and main text as suggested. We did not adjust the main title because we did not believe it overplayed the paper's important findings. Whilst in this modest study, the post-hoc analysis above did not show significant advantages of adjusting for LVM versus adjusting for sex, within each sex subgroup, it is likely that the improvement in the model adjusting for sex is mediated by its strong relationship with LVM rather than the other way around. An individual's precision is likely higher when data are available.

Page 3, line 72

Whilst the improvement of the model by the inclusion of LVM is mechanistically insightful, the inclusion of sex, which strongly associates with LVM, proved promising as a pragmatic alternative. Use of this score as a triage tool for CT screening shows potential to address the challenges of scale and equity, as non-invasive imaging technology continues to advance. The use of a cut-off on the hs-cTnI≥2.3 is predicted to decrease the number of scans required to detect clinically actionable CAD, while maintaining high diagnostic performance, particularly in low-risk or SMuRF-less patients by up to two-fold.
`

Page 18, line 399

Although the demonstrated value of LVM adjustment is consistent with the hypothesis that myocardial volume contributes to low-level hs-cTnI release, a simpler hs-cTnI-by-sex model performed similarly to the hs-cTnI-by-LV mass model. This likely relates to the very strong relationship of sex and LVM (noting that when sex was included in LVM in the model, it was not a significant contributor). This has practical implications, suggesting that sex and BMI/BSA could be used to replace LVM in clinical settings where LVM is unavailable.
